# The deep Earth origin of the Great Unconformity

**DOI:** 10.1038/s41586-026-11156-5

**Published:** 2026-11-04

**Authors:** Santosh Dhungana, Nicolas Flament

**Affiliations:** Environmental Futures, School of Science, University of Wollongong, Wollongong, New South Wales Australia

**Keywords:** Geodynamics, Tectonics, Sedimentology

## Abstract

The Great Unconformity marks a globally extensive erosive surface linked to climatic shifts^1^ and environmental transitions^2^ that preceded the Cambrian explosion of life. The processes proposed to explain the formation of the Great Unconformity include glacial erosion during the Snowball Earth^1,3,4^, rifting and plume activity^5–7^, sea-level fall^8^ and mantle-driven dynamic topography^6,9–11^. However, the dynamics of these processes have not been tested. Here we show that continental-scale uplift and subsidence associated with long-wavelength dynamic topography^12^ due to mantle convection drove the formation and preservation of the Great Unconformity. Our models predict prolonged continental-scale Neoproterozoic uplift and long-term Phanerozoic subsidence for Laurentia, Baltica and North China. This vertical motion scenario is ideal for the formation and preservation of the Great Unconformity, which is a first-order surface expression of deep Earth processes. The predicted prolonged continental uplift throughout Cryogenian times would have resulted in marked erosion due to the action of glaciers^13^. The large area of Laurentia and limited deformation at its margins explain why the Great Unconformity is best preserved and documented there. Our models predict cycles of dynamic topography that are broadly reflected in preserved sediment volume across continents^1,14^, and open new avenues for understanding the environments of Cambrian animal diversification.

## Abstract (web summary)

Continental-scale uplift and subsidence associated with long-wavelength dynamic topography due to mantle convection drove the formation and preservation of the Great Unconformity.

## Main

**Fig. 1:**
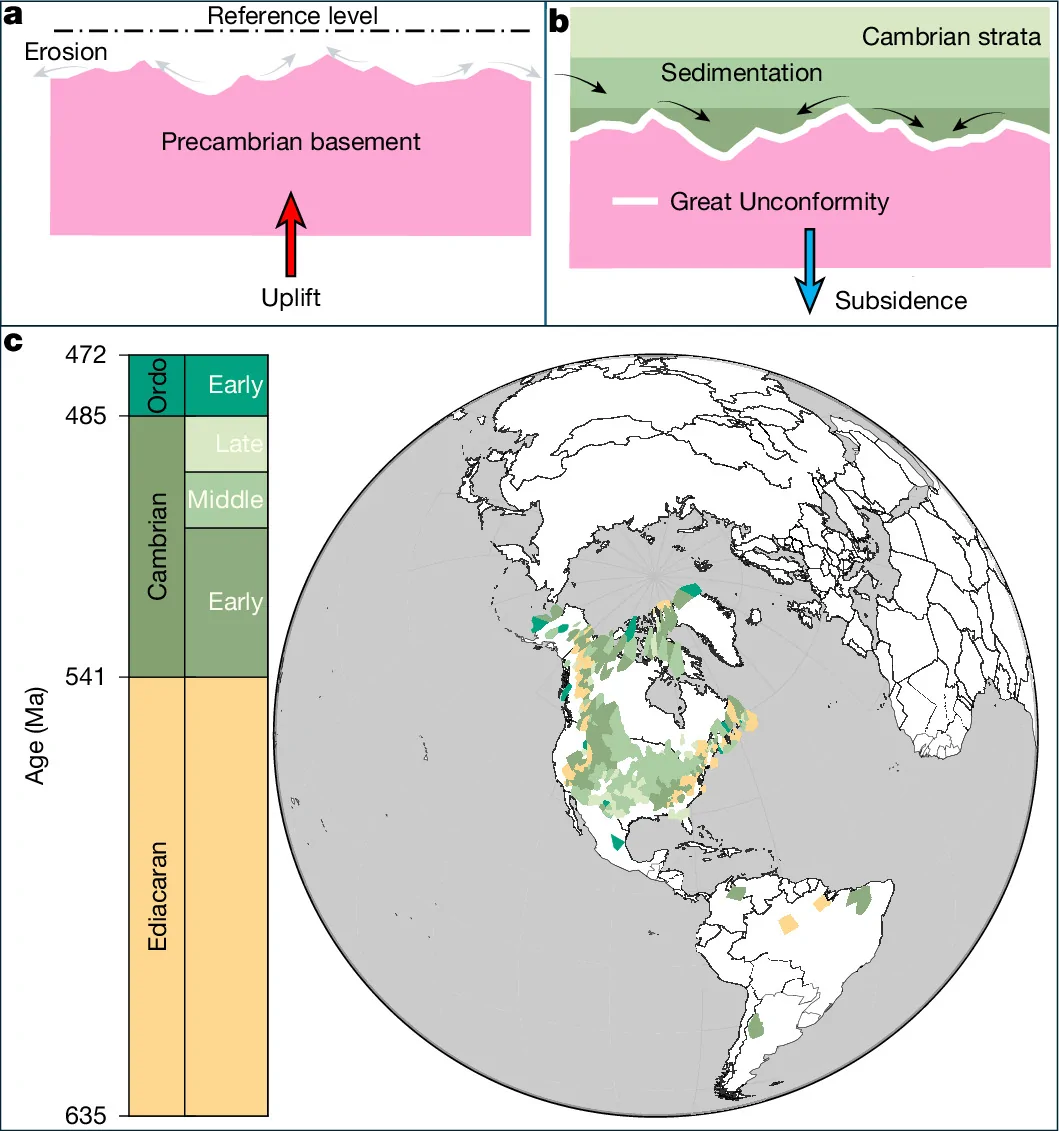
Definition and inferred spatial distribution of the Great Unconformity. **a**,**b**, The Great Unconformity was formed by continental-scale erosion of Precambrian crystalline basement (**a**) followed by Cambrian sedimentation (**b**). In **b**, the sedimentary strata are coloured by age and the white line represents the Great Unconformity surface. The schematics in **a** and **b** are not to scale. **c**, Spatial distribution of the oldest sedimentary strata deposited between Late Ediacaran and Early Ordovician (Ordo) times in the database Macrostrat^55^, coloured by age. Lambert azimuthal equal-area projection at the surface of Earth.

An unconformity is a surface that represents a temporal gap (hiatus) in the geologic record in which younger sediments are deposited over older rocks, after a period of erosion or non-deposition^15^. Although unconformities are common in the rock record, they markedly vary in spatial extent and hiatus duration. The largest known unconformity is called the Great Unconformity^16^. It represents an erosional or non-depositional hiatus lasting about 200 million years (Myr) or longer, and marks the onset of sedimentation at the start of Phanerozoic time and of the Cambrian explosion of life^2^. The Great Unconformity was first described in the Grand Canyon^17^, and has been established across North America from a digital database of sedimentary deposits^1,2,9^ and thermochronological data^3,4,6,7,10,18^. The oldest sediments at the base of the Great Unconformity in North America are between 635 Myr old and 482 Myr old (Ediacaran to Early Ordovician times; Fig. 1). Spatially extensive unconformities of a similar age have been documented in Baltica^19–24^ and in North China^8,25–28^. The global sedimentary volume estimated from geologic maps and borehole data more than doubled across the Precambrian–Phanerozoic boundary^1,14^.

The cause or causes of the Great Unconformity are unknown. The proposed mechanisms include extensive glacial erosion during Snowball Earth (about 720–635 Myr ago or Ma)^1,3,4^, rifting and plume activity^5–7^, sea-level fall^8^ and mantle-driven dynamic topography^6,9–11^. These processes have not been dynamically tested, with limited comparison between global models and global datasets to explain the spatial distribution of the Great Unconformity. Here, we propose that continental-scale dynamic topography^6,9–12,29,30^, which is transient over thousands of kilometres and tens of millions of years^12^, provided the driving force for surface processes to generate the continental-scale denudation and sedimentation consistent with the geologic record of the Great Unconformity^6,9–11^. Dynamic uplift followed by dynamic subsidence can produce large-scale erosional surfaces and unconformities^11,12,31^. We test this hypothesis by analysing the evolution of continental dynamic topography predicted by a series of mantle convection models, and comparing it with the volume of sediment preserved on continents over time^1,14^.

## Mantle convection and dynamic topography

**Extended Data Fig. 1:**
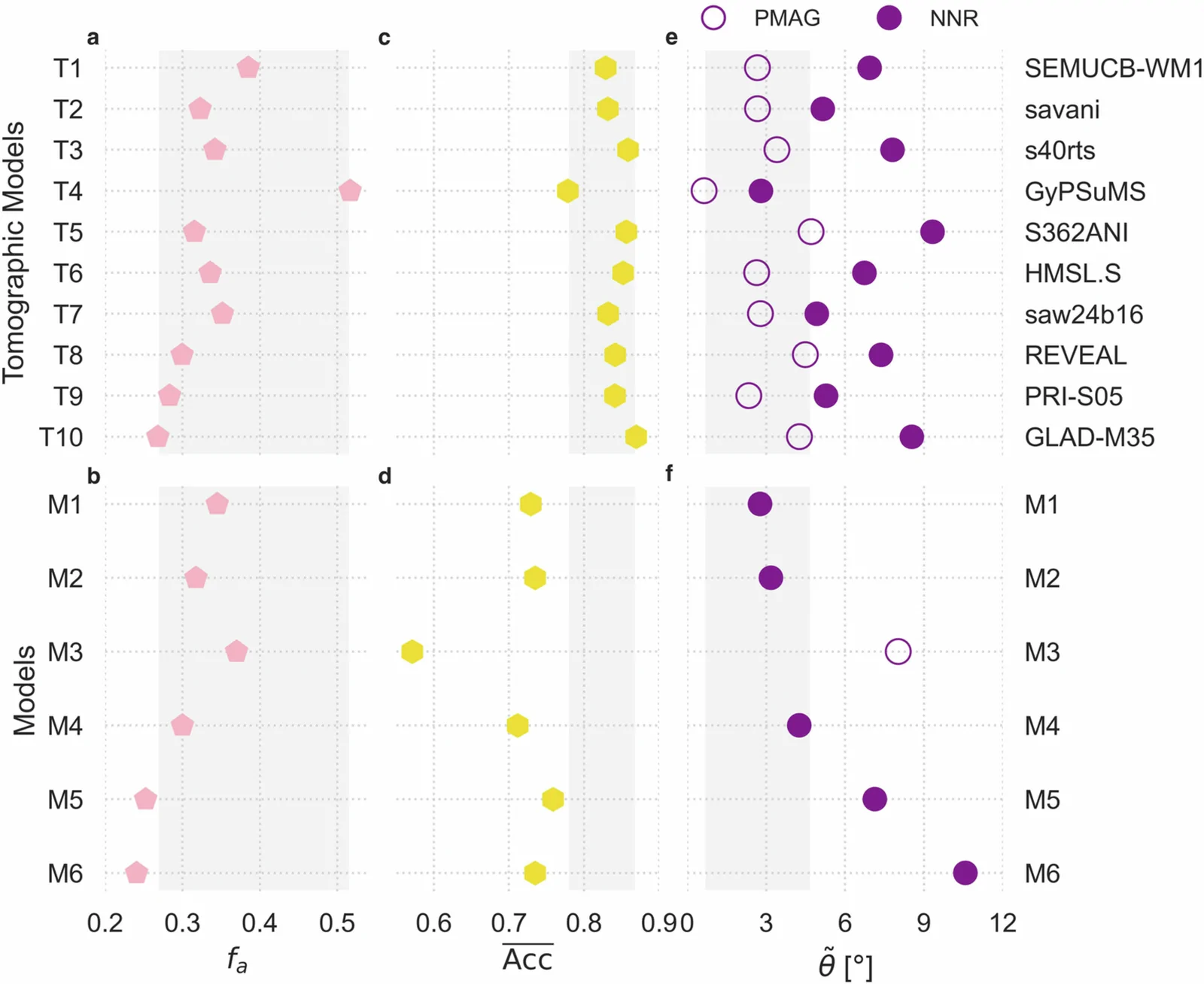
Spatial match between BLOBSs predicted by mantle flow models and LLSVPs inferred from S-wave tomographic models^78–87^ and BLOBS and LLSVP proximity to volcanic eruptions. **a-b**, Fractional area *f*_*a*_ of deep mantle cluster maps covered by LLSVPs in tomographic models (**a**) and by BLOBSs in flow models at present day (**b**). **c-d**, Average spatial match ($\overline{\mathrm{Acc}}$ between (**c**) LLSVPs for a given tomographic model and LLSVPs from the other nine tomographic models, and (**d**) present-day BLOBSs for a given mantle flow model and LLSVPs averaged from the ten tomographic models. **e-f**, Time-averaged median of minimum angular distances $\widetilde{\theta }$ between LLSVPs and volcanic eruptions (**e**) and between BLOBSs and volcanic eruptions (**f**) from 320 Ma. In **a-f**, the first row shows results for ten tomographic models (stationary LLSVPs), and the last row shows results for six flow models (mobile BLOBSs) across which parameters are varied as indicated. ‘T1-T10’: tomographic models 1−10; ‘M1-M6’: mantle flow model Cases 1−6. The grey shadings in **a**-**f** highlight the range of results for tomographic models.

To investigate the deep Earth origin of the Great Unconformity, we considered palaeogeographically constrained mantle convection models from 1 billion years ago (Gyr ago or Ga). Input parameters were varied across a series of six models M1–M6 (Extended Data Table 1). These models were driven by tectonic reconstructions in the no-net-rotation (NNR) frame of reference, except for M3, which was based on a palaeomagnetic (PMAG) frame of reference. As in previous studies^32,33^, we first verified that the big lower-mantle basal structures (BLOBSs) predicted by the models were consistent with large low-shear velocity provinces (LLSVPs) inferred from 10 tomographic models and that reconstructed volcanic provinces were above or close to the mobile BLOBSs predicted by models between 320 Ma and 0 Ma (Methods and Extended Data Fig. 1). Model M1 was preferred because it predicted BLOBS areas and locations consistent with LLSVPs, and volcanic provinces close to BLOBS edges over time (Extended Data Fig. 1).

**Fig. 2:**
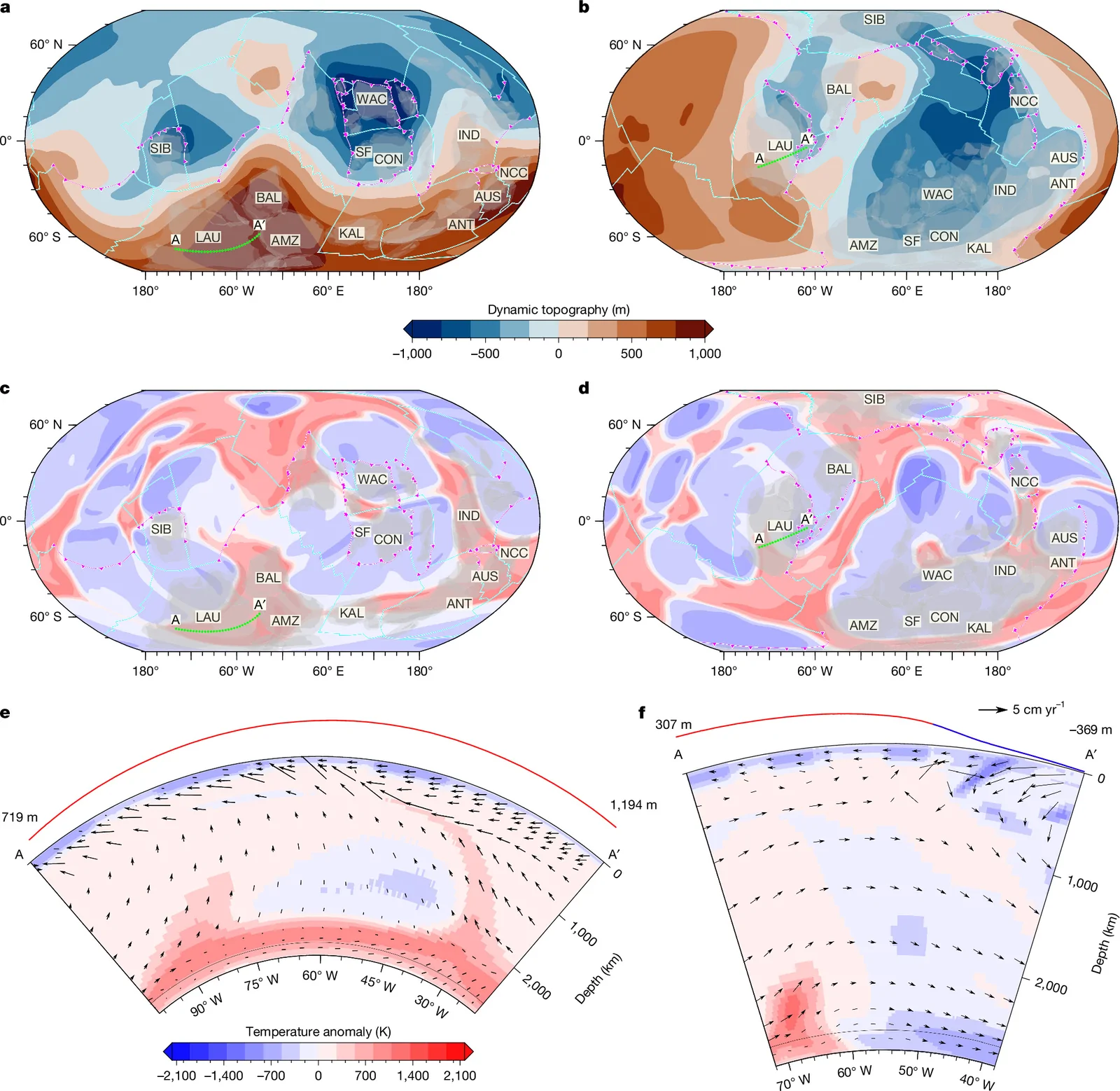
Predicted joint evolution of dynamic topography and mantle temperature. **a**,**b**, Dynamic topography at 640 Ma (**a**) and 440 Ma (**b**). **c**,**d**, Mantle temperature anomaly at 2,677 km depth at 640 Ma (**c**) and 440 Ma (**d**). In **a**–**d**, cyan lines represent mid-ocean ridges and transform faults, and magenta lines represent subduction zones with triangles on the overriding plate. Transparent grey polygons represent continental blocks, with labels as follows: AMZ, Amazonia; ANT, Antarctica; AUS, Australia; BAL, Baltica; CON, Congo; IND, India; KAL, Kalahari; LAU, Laurentia; NCC, North China; SF, São Francisco; SIB, Siberia; and WAC, West Africa. The green lines with diamonds are great circle transects A–A′ shown in **e** and **f**. The length of the great circle depends on its latitudinal position. Robinson projection at the surface of Earth. **e**,**f**, Cross-sections of temperature anomalies and dynamic topography across Laurentia (transect A–A′) at 640 Ma (**e**) and 440 Ma (**f**). Polar projections of longitude and depth. The colour bar in **e** is the temperature anomaly scale for **c**–**f**. Black arrows are velocity vectors. The amplitude of dynamic topography (not to scale) is represented above the cross-sections with positive amplitudes in red, negative amplitudes in blue, and reported values at A and A′. The black solid line indicates a depth of 2,667 km.

We investigated the dynamic topography predicted by these mantle flow models, which depends on the distribution of buoyancy sources and viscosity. Sources of buoyancy closer to the surface have a greater influence on the amplitude of dynamic topography, and contributions from the deep mantle are largest at the longest wavelengths^34,35^. We computed dynamic topography from sources of buoyancy and viscosity variations deeper than 350 km depth^30^, so that sources of dynamic topography nearer the surface (for example, ref. ^36^) were neglected. This setup resulted in smooth dynamic topography (Fig. 2a,b) at the continental scale^30,37^, which was desirable to test the hypothesis that the Great Unconformity could be linked to mantle flow. However, the setup is known to overpredict the amplitude of long-wavelength dynamic topography by a factor of about 2 (refs. ^30,37^). We therefore focused on the trend of dynamic topography evolution, which was key to our analysis, rather than its amplitude.

The motion of tectonic plates and subduction zones affected mantle convection and dynamic topography^12,29,32,33,37,38^ (Fig. 2 and Supplementary Videos 1 and 2). At 640 Ma, dynamic topography exhibited a north–south hemispheric planform with positive dynamic topography beneath Laurentia, Baltica, North China, Amazonia, Australia, the Kalahari Craton and Antarctica (Fig. 2a), which were above a BLOBS in the absence of subduction south of 50° S (Fig. 2c). Laurentia was tilted down to the southwest, positioned above whole-mantle upwelling (Fig. 2e). The fast motion of continental blocks and of subduction zones between 600 Ma and 320 Ma (ref. ^39^) reorganized mantle convection (Supplementary Videos 1 and 2) so that by 440 Ma, the hemispheric planform of dynamic topography was oriented east–west, with negative dynamic topography beneath most continental blocks, including at high latitude in both hemispheres (Fig. 2b). Laurentia was tilted down to the southeast, because of subduction-driven downwelling along its eastern margin and whole-mantle upwelling above a BLOBS in the west (Fig. 2f).

## Mapping the predicted Great Unconformity

**Fig. 3:**
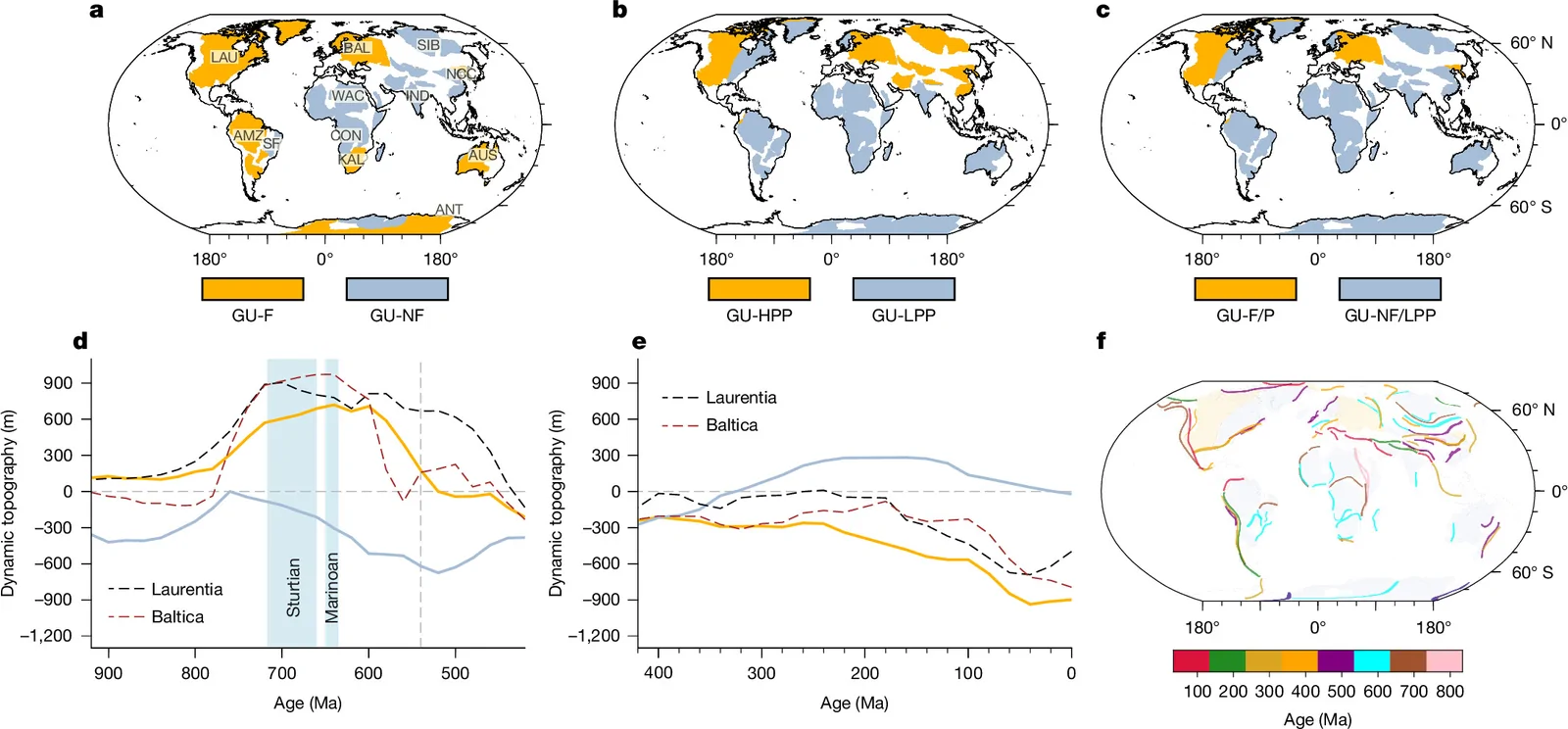
Spatiotemporal evolution of dynamic topography. **a–c**, Spatial distribution of two clusters across continental blocks that existed from 920 Ma: GU-F (Great Unconformity—formation) and GU-NF (Great Unconformity—no formation) between 920 Ma and 420 Ma (**a**); GU-HPP (Great Unconformity—high preservation potential) and GU-LPP (Great Unconformity—low preservation potential) from 420 Ma (**b**); regions in which the Great Unconformity was formed and preserved (GU-F/P), as opposed to regions with low formation and preservation potential (GU-NF/LPP) (**c**). Robinson projection at the surface of Earth. **d**,**e**, Temporal evolution of averaged continental dynamic topography for each cluster in **a** and **b**, respectively. The black and brown dashed lines represent the average dynamic topography for Laurentia and Baltica, respectively. The vertical dashed line represents the start of Cambrian times, and the horizontal dashed line represents the datum for dynamic topography. **d**, Averaged continental dynamic topography for clusters GU-F (orange) and GU-NF (grey) between 920 Ma and 420 Ma. The light-blue-shaded regions represent the duration of the two main glaciations during Cryogenian times. **e**, Averaged continental dynamic topography for clusters GU-HPP (orange) and GU-LPP (grey) from 420 Ma. **f**, Locations of orogens^56^ from 900 Ma (coloured by age in 100 Ma intervals). Robinson projection at the surface of Earth.

**Extended Data Fig. 2:**
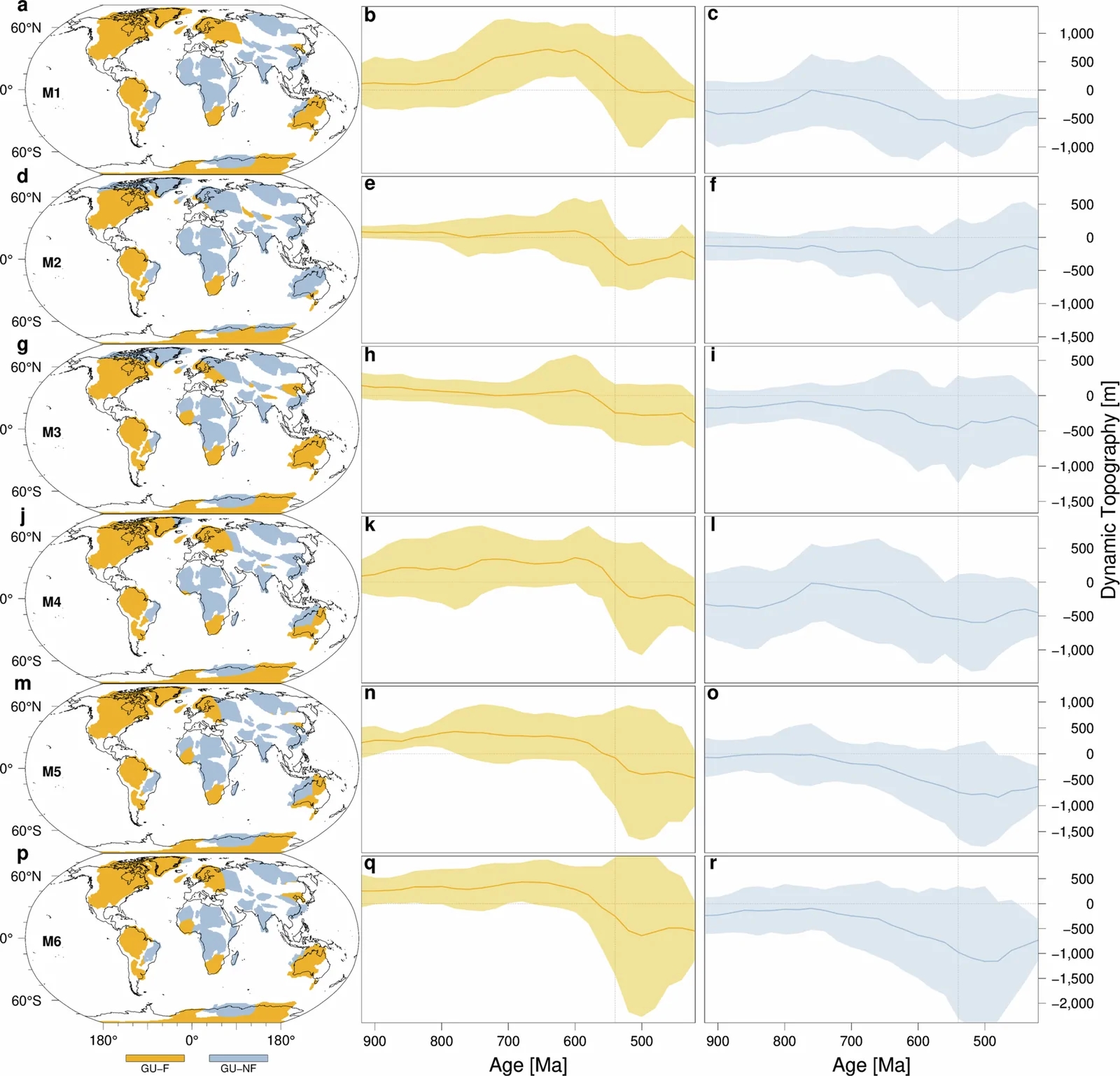
Cluster analysis of dynamic topography between 920–420 Ma for all models. Spatial distribution of two clusters GU–F (‘Great Unconformity – Formation’) and GU–NF (‘Great Unconformity – No Formation’) revealed by *k*-means cluster analysis showing continental blocks that existed from 920 Ma in their present-day location (**a,d,g,j,m,p**). Robinson projection at the surface of Earth. Temporal evolution of averaged continental dynamic topography for each cluster in (**a,d,g,j,m,p**) with ± 2 standard deviation envelopes (**b-c,e-f,h-i,k-l,n-o,q-r**). The vertical dashed grey line in (**b,e,h,k,n,q**) is the start of Cambrian times, and the horizontal dashed lines the datum for the dynamic topography. M1–M6 denote the six considered model cases.

We used *k*-means cluster analysis to quantify the spatial and temporal evolution of continental dynamic topography during the formation (920–420 Ma) and preservation (420–0 Ma) of the Great Unconformity (Fig. 3 and Methods). We established that considering two clusters for each period was prototypical for most considered model cases except M3 and M5, for which three clusters were appropriate (Methods and Supplementary Fig. 1). These clusters, which we represented in the present-day continental configuration, captured regions in which dynamic topography evolved similarly over time (Fig. 3 and Extended Data Fig. 2).

**Extended Data Fig. 3:**
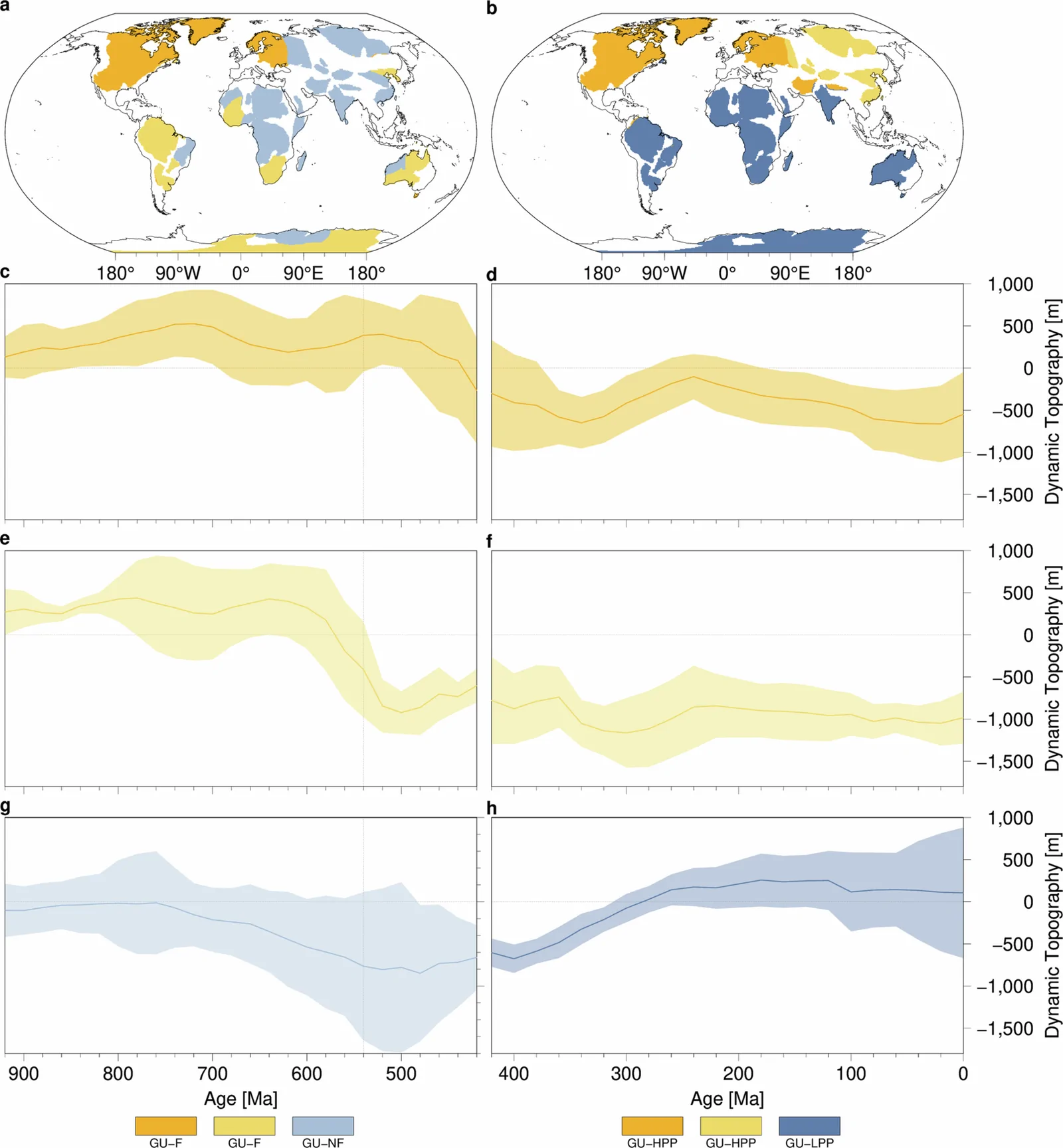
Cluster analysis of dynamic topography for model M5 between 920–420 Ma and 420–0 Ma. **a**-**b**, Spatial distribution of three clusters revealed by *k*-means cluster analysis between (**a**) 920–420 Ma and (**b**) 420–0 Ma. Robinson projection at the surface of Earth. **c**-**h**, Temporal evolution with ± 2 standard deviation envelopes for spatial cluster in **a-b** for 920–420 Ma (**c,e,g**) and 420–0 Ma (**d,f,h**) for continental blocks that existed from 920 Ma in their present-day location. The clusters are classified as two GU–F (‘Great Unconformity – Formation’) clusters and one GU–NF (‘Great Unconformity – No Formation’) clusters for 920–420 Ma (**a**) and two GU–HPP (‘Great Unconformity – High Preservation Potential’) clusters and one GU–LPP (‘Great Unconformity – Low Preservation Potential’) cluster for 420–0 Ma (**b**). The vertical dashed grey line in **c,e,g** is the start of Cambrian times, and the horizontal dashed line in **c-h** the datum for the dynamic topography.

For the period 920–420 Ma, the main continental blocks in cluster GU-F (Great Unconformity—formation) were Laurentia, Baltica, northeastern North China, most of South America, Western Australia, the Kalahari Craton and parts of Antarctica. Cluster GU-NF (Great Unconformity—no formation) spanned most of Africa, Asia, parts of Antarctica and the São Francisco Craton (Fig. 3a). Cluster GU-F captured positive dynamic topography (Fig. 3d), mainly above whole-mantle upwelling (Supplementary Video 1). Dynamic topography was relatively stable between 900 Ma and 800 Ma, increased rapidly between 800 Ma and 720 Ma, followed by a gradual increase between 720 Ma and 600 Ma, and a rapid decrease between 600 Ma and 420 Ma (Fig. 3d). The predicted uplift would have promoted glacial erosion^1,13^ during the proposed Sturtian and Marinoan global glaciations (Fig. 3d). The GU-F pattern of prolonged dynamic uplift during Neoproterozoic times (Fig. 2 and Supplementary Videos 1 and 2), followed by dynamic subsidence during Cambrian times (Fig. 3d) was consistent across all mantle convection models (Extended Data Figs. 2 and 3a,c,e). Cluster GU-NF captured dynamic topography on average negative throughout 920–420 Ma with gradual increase between 900 Ma and 760 Ma, a rapid decline between 760 Ma and 520 Ma, and renewed increase between 520 Ma and 420 Ma (Fig. 3d). In cluster GU-NF, Siberia and the Congo and West African cratons were in a dynamic topography low surrounded by subduction zones (Fig. 2a,c and Supplementary Videos 1 and 2).

For the period 420–0 Ma, cluster GU-HPP (Great Unconformity—high preservation potential) spanned Western Laurentia, Baltica, North China and Siberia, and cluster GU-LPP (Great Unconformity—low preservation potential) spanned Eastern Laurentia, Africa, Australia, Antarctica and India (Fig. 3b). The dynamic topography for GU-HPP, which was generally negative because of subduction (Supplementary Video 1), remained relatively stable between 420 Ma and 240 Ma, before gradually declining until 0 Ma (Fig. 3e). By contrast, in GU-LPP dynamic topography increased between 420 Ma and 240 Ma, was stable between 240 Ma and 120 Ma, then gradually declined until 0 Ma (Fig. 3e).

**Extended Data Fig. 4:**
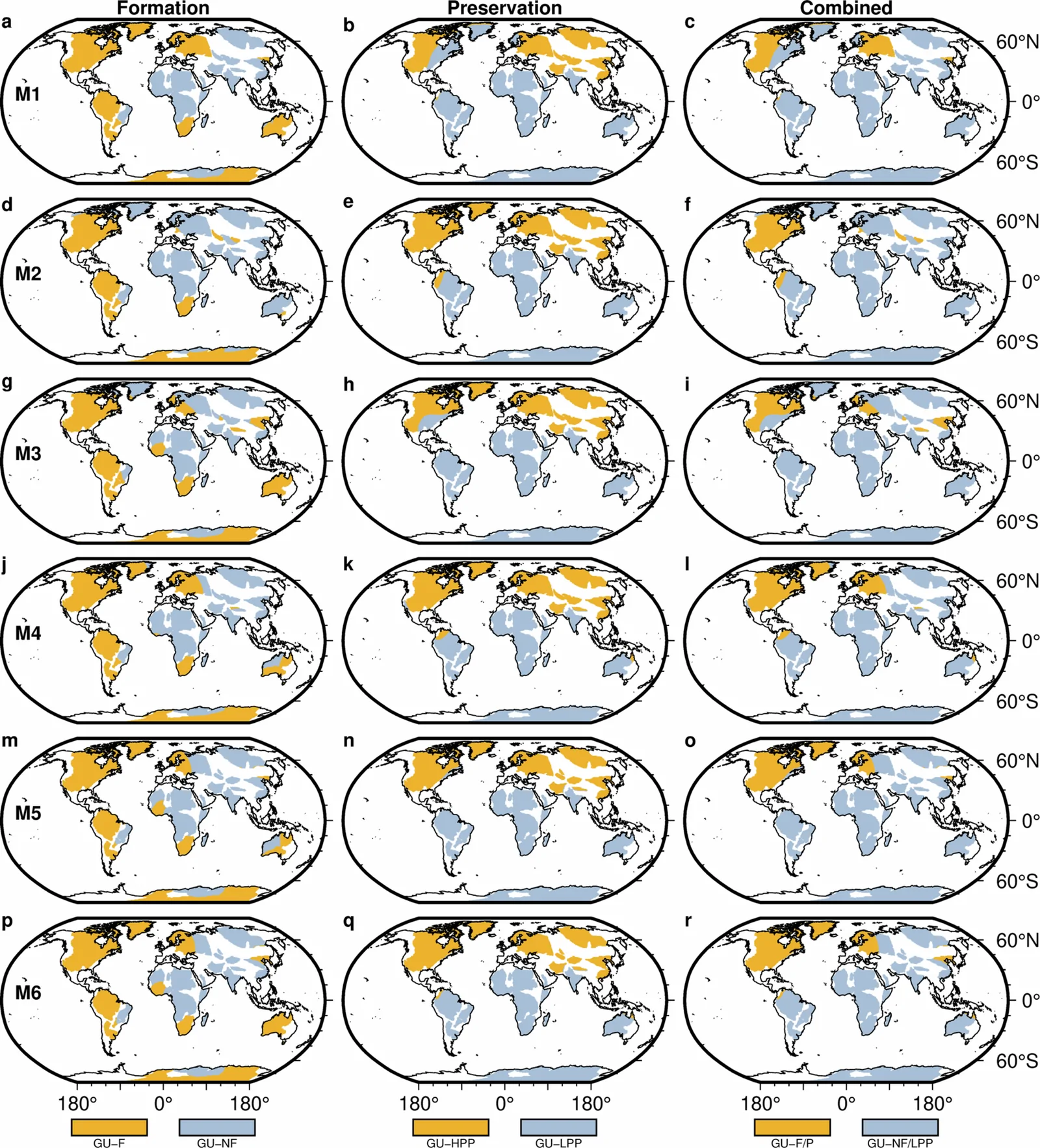
Spatial distribution of dynamic topography clusters for all models during the Great Unconformity formation (920–420 Ma) and preservation (420–0 Ma) periods. **a**-**r**, Spatial distribution of two clusters revealed by *k*-means clustering for continental blocks that existed from 920 Ma in their present-day configuration with GU–F and GU–NF for period of 920–420 Ma (**a,d,g,j,m,p**), GU–HPP and GU–LPP between 420–0 Ma (**b,e,h,k,n,q**) and regions forming and preserving the Great Unconformity (GU–F/P) and regions with low preservation potential (GU–NF/LPP) (**c,f,i,l,o,r**) obtained by combining ‘Formation’ and ‘Preservation’ maps. Robinson projection at the surface of Earth. M1-M6 denote the six considered model cases.

**Extended Data Fig. 5:**
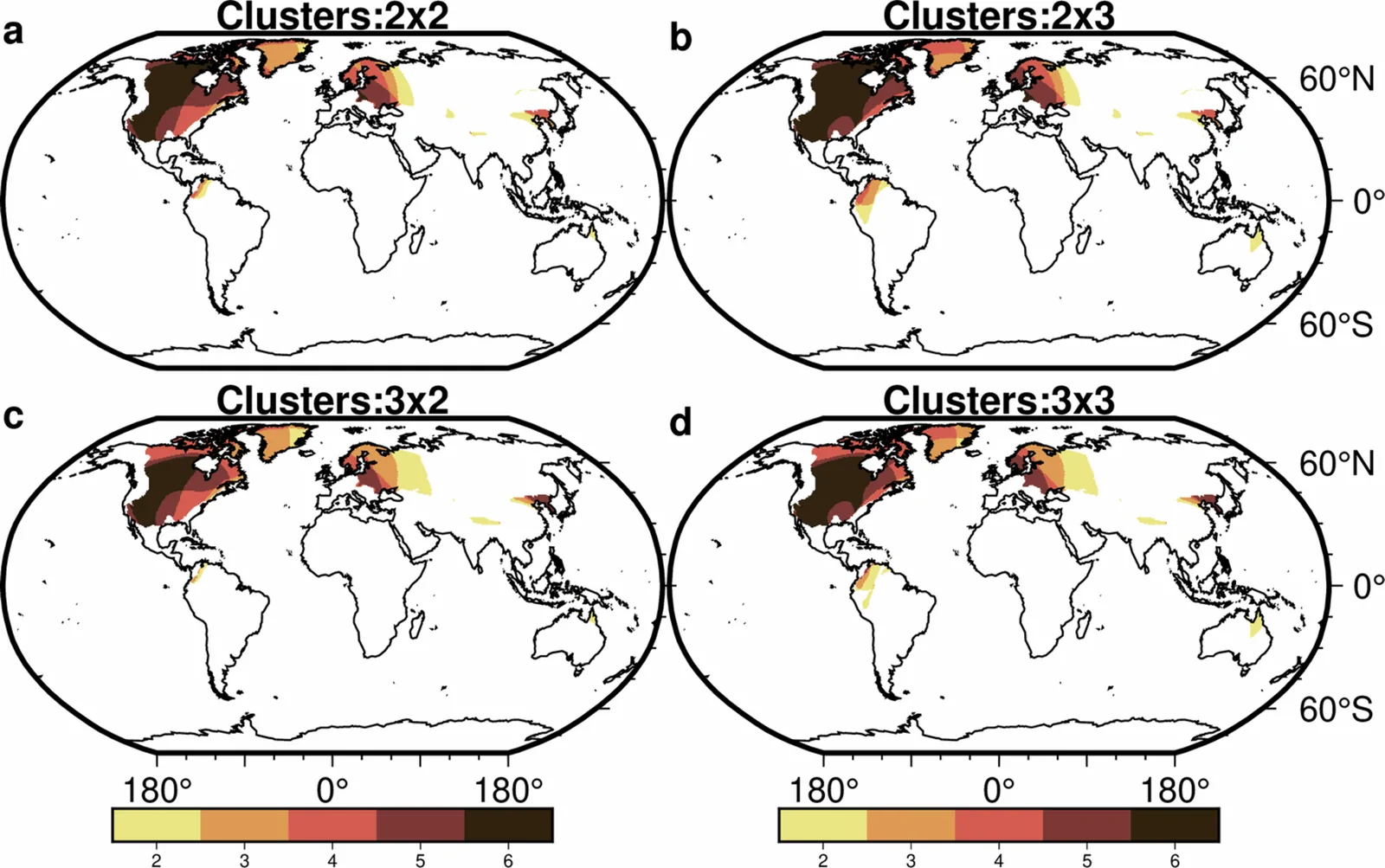
Vote maps showing spatial regions forming and preserving the Great Unconformity across all model and cluster combinations. **a**-**d**, Maps showing the areas in which the Great Unconformity is predicted to have formed between 920–420 Ma and been preserved between 420–0 Ma using **a**, two clusters for each period; **b**, two clusters for 920–420 Ma and three clusters for 420–0 Ma; **c**, three clusters for 920–420 Ma and two clusters for 420–0 Ma; and **d**, three clusters for 920–420 Ma and three clusters for 420–0 Ma. Colours indicate model agreement (‘vote map’), with a score equal to six indicating consensus across all models for a region. Robinson projection at the surface of Earth.

We derived a map highlighting the areas in which the Great Unconformity was predicted to have formed and to have a high preservation potential, which consisted of Laurentia, Baltica and northeastern North China for model M1 (Fig. 3c) and included areas around Laurentia and Baltica in all six models (Extended Data Figs. 3 and 4). We analysed the sensitivity of these results to the number of clusters (either two or three) for each period (formation 920–420 Ma, preservation from 420 Ma) from vote maps (Methods) of areas in which the Great Unconformity was predicted to exist across the six models (Extended Data Fig. 5). This confirmed that Laurentia, Baltica and North China were the main continental blocks in which the Great Unconformity was predicted to have formed and to be preserved, with northern South America emerging as a possible additional region (Extended Data Fig. 5).

## Dynamic topography and sediment flux

**Fig. 4:**
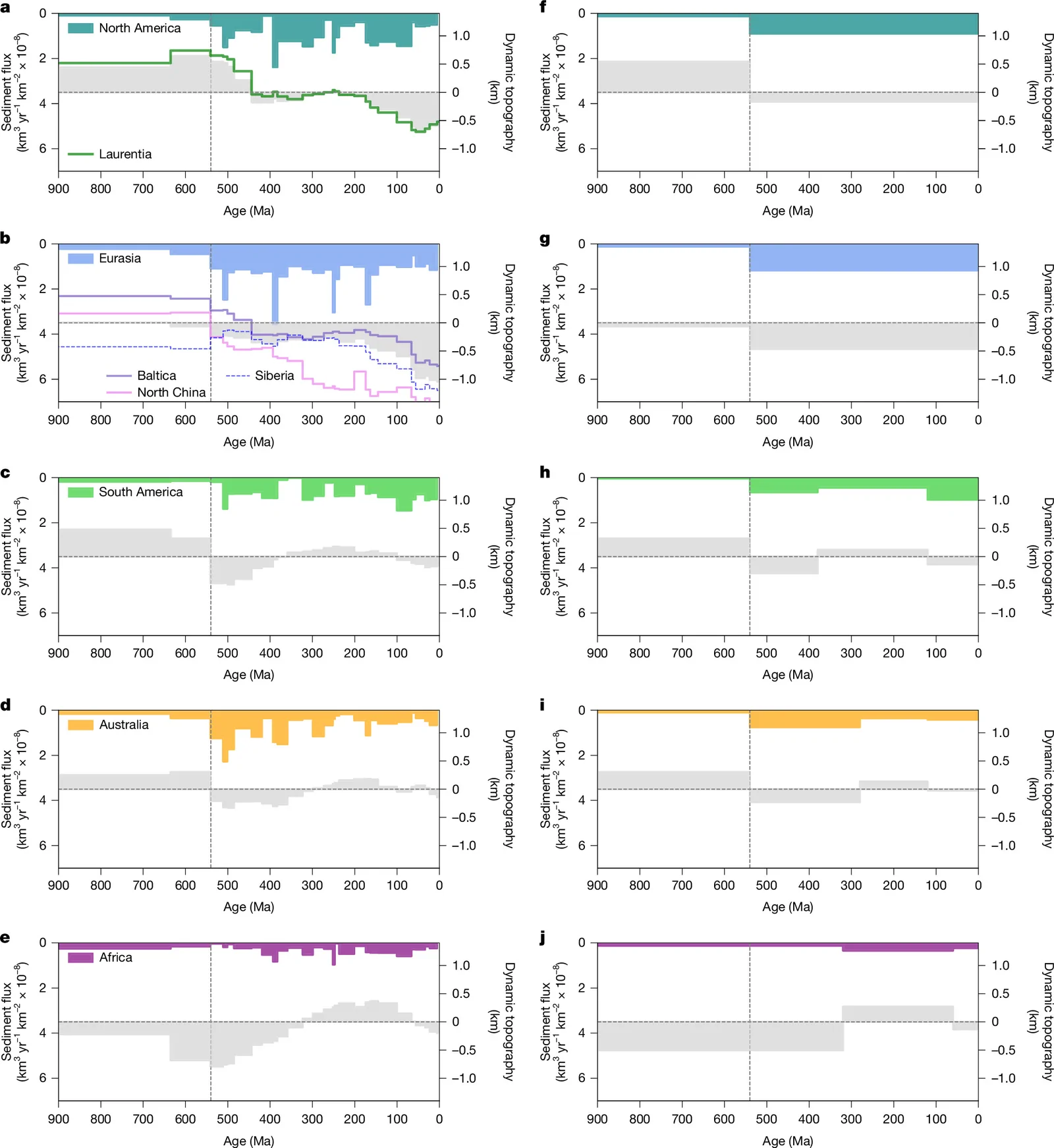
Volumetric flux of preserved sediments and average dynamic topography for each continent. **a–e**, Evolution of the preserved volume of sediment per year and per unit continental area (increasing down) (left *y* axis, coloured bar charts) and dynamic topography (right *y* axis, grey bar charts and coloured lines) for each continent averaged at time steps corresponding to the sediment preservation data^1,14^. Coloured solid and dotted lines represent the evolution of dynamic topography for Laurentia and continental blocks in Eurasia. **f–j**, Data in **a–e** temporally averaged for dynamic topography cycles in each continent. In each panel, the vertical dashed line represents the start of Cambrian times, and the horizontal dashed line represents the datum for dynamic topography.

**Extended Data Fig. 6:**
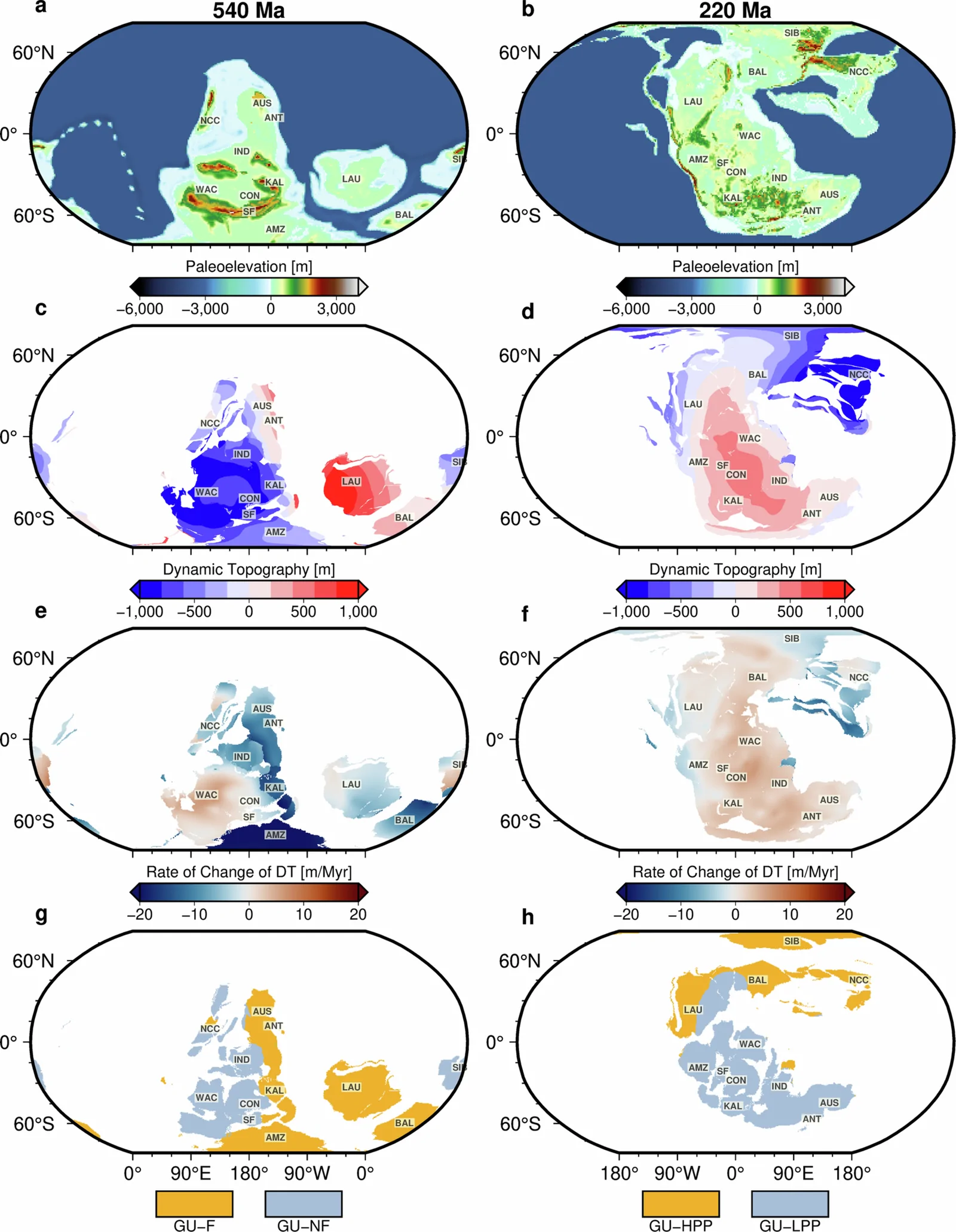
Paleo-elevations, dynamic topography, dynamic topography rate of change and clusters reconstructed using the PALEOMAP rotations ^99^. **a**-**b**, Paleo-elevation maps from ref. ^99^ at 540 Ma (**a**) and 220 Ma (**b**). **c**-**f**, Predicted dynamic topography and long-term dynamic topography rate of change reconstructed at 540 Ma (**c**,**e**) and 220 Ma (**d**,**f**). **g**-**h**, Dynamic topography clusters (**g**) at 540 Ma and at 220 Ma (**h**). In **a**-**h** abbreviated labels indicate ‘AMZ’: Amazonia, ‘ANT’: Antarctica, ‘AUS’: Australia, ‘BAL’: Baltica, ‘CON’: Congo, ‘IND’: India, KAL: Kalahari, LAU: Laurentia, ‘NCC’: North China, ‘SF’: São Francisco, ‘SIB’: Siberia and ‘WAC’: West Africa. Robinson projection at the surface of Earth.

**Extended Data Fig. 7:**
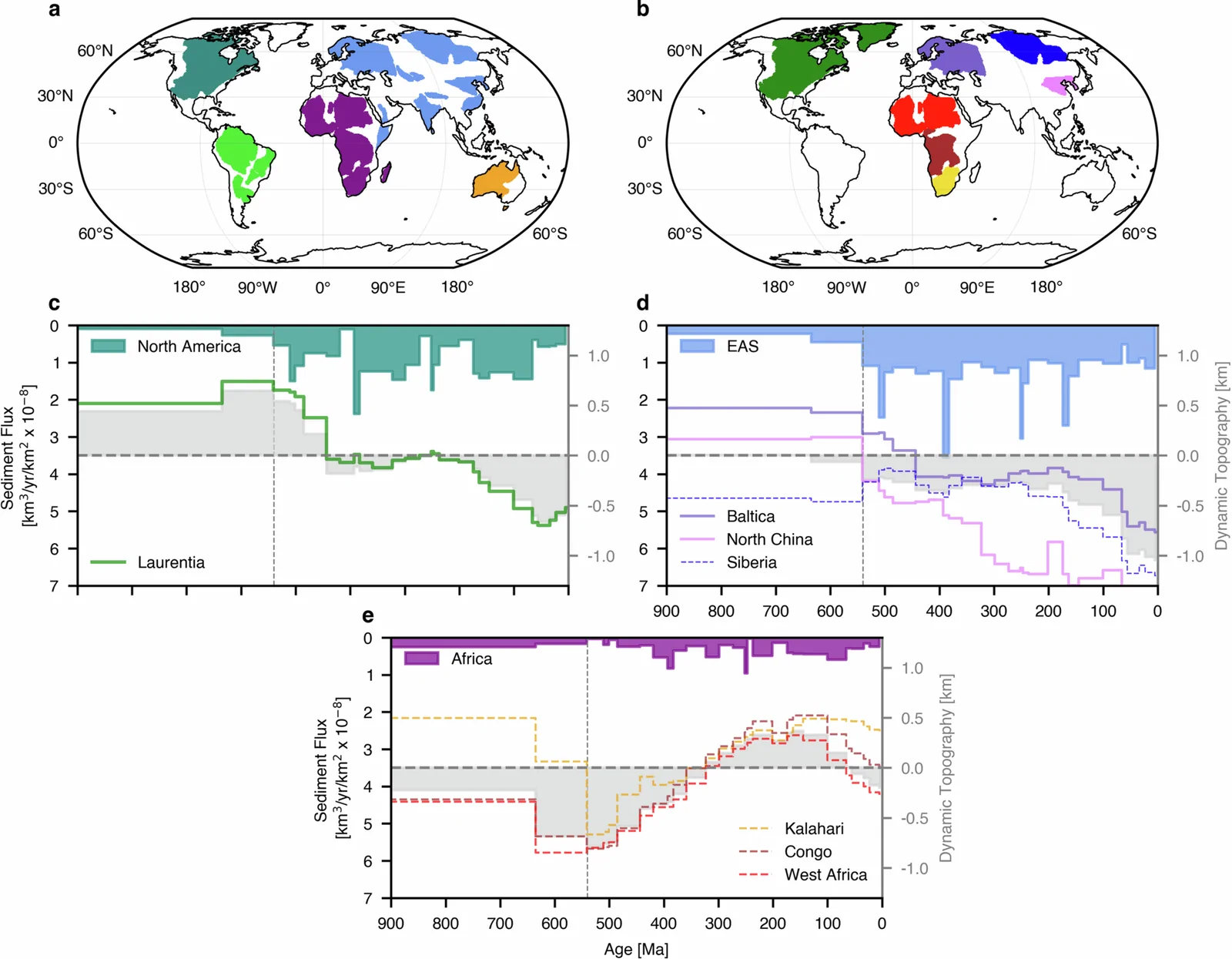
Preserved sediments flux and dynamic topography across continents and continental blocks. **a**, Continental blocks considered to determine preserved sediment flux^1,14^. **b**, Selected continental blocks continuously existing from 920 Ma. **c**-**e**, Temporal evolution of the sediment volume per year and per unit continental area (increasing down), and average dynamic topography across North America (**c**), Eurasia (**d**) and Africa (**e**). The coloured solid and dotted lines represent the temporal evolution of dynamic topography for the continental blocks shown in (**b**).

**Extended Data Fig. 8:**
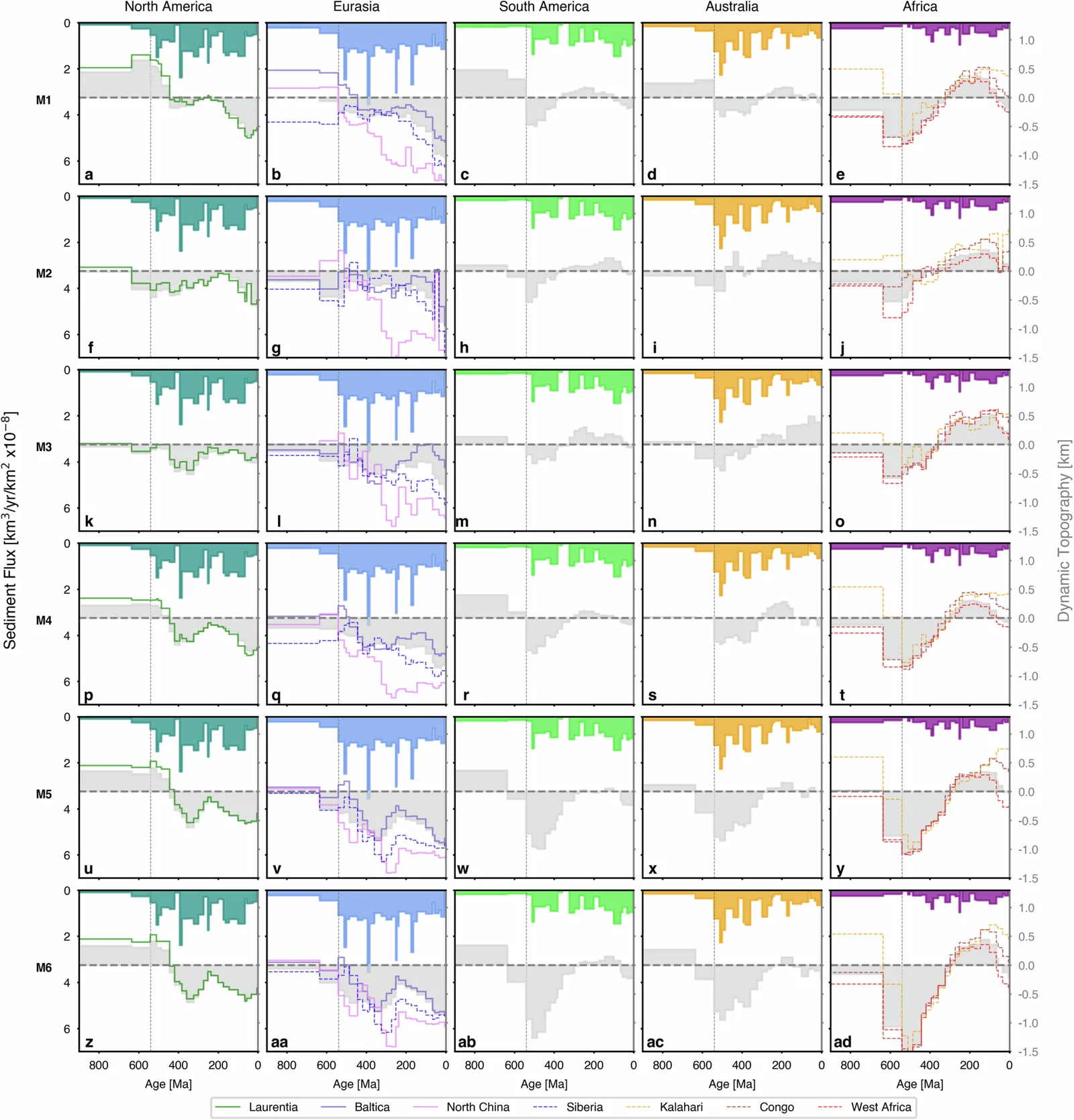
Temporal evolution of volumetric flux of preserved sediments compiled by ref. ^14^ (obtained from ref. ^1^) and average dynamic topography cycles across continents between 920–0 Ma for all mantle flow models. **a**-**ad**, Temporal evolution of preserved volume of sediment per year and per unit continental area (increasing down), and dynamic topography for each of six considered model cases (M1–M6) individually averaged at time steps corresponding to the sediment preservation data^1,14^ across Laurentia (**a,f,k,p,u,z**), Amazonia (**b,g,l,q,v,aa**), Australia (**c,h,m,r,w,ab**), Eurasia (**d,i,n,s,x,ac**) and Africa (**e,j,o,t,y,ad**). Coloured solid and dotted lines indicate the temporal evolution of dynamic topography for selected continental blocks in Eurasia and Africa.

The dynamic topography evolution predicted by our models was consistent with the preserved sediment flux for each continent except Africa^1,14^ (Fig. 4 and Methods). Predicted prolonged Precambrian uplift, followed by rapid Cambrian subsidence, generally matched increased sediment flux across the Precambrian–Cambrian boundary (Fig. 4a–e). A shift from erosion to sedimentation reflecting decreasing dynamic topography (Fig. 4) and flooded continents (Extended Data Fig. 6a) was predicted for all continents except for Eurasia and Africa, which consisted of tectonic blocks with contrasting Neoproterozoic histories. In Eurasia, dynamic uplift was predicted for Baltica and North China, and dynamic subsidence for Siberia^40^ (Fig. 4b and Extended Data Figs. 7d and 8b,g,l,q,v,aa). In Africa, dynamic uplift was predicted for the Kalahari Craton, and dynamic subsidence for the Congo and West African cratons (Extended Data Figs. 7e and 8e,j,o,t,y,ad) was consistent with their sedimentary record of long-lived depocentres^41,42^. Extensive Precambrian orogens within Africa enhanced erosion and sedimentation (Fig. 3f and Extended Data Fig. 6), leading to a landscape in which negative dynamic topography did not result in increased sediment flux at the base of the Cambrian (Extended Data Fig. 6a,c,e,g).

The evolution of predicted Phanerozoic dynamic topography differed across continental blocks. Laurentia, Baltica and North China were characterized by sustained dynamic subsidence, which aligned with increased preserved sediment flux across North America and Eurasia (Fig. 4). Conversely, predicted renewed dynamic uplift of South America and of Australia, which would have eroded earlier unconformities^11,31^ and formed new ones, corresponded to decreased preserved sediment flux (Fig. 4).

**Extended Data Fig. 9:**
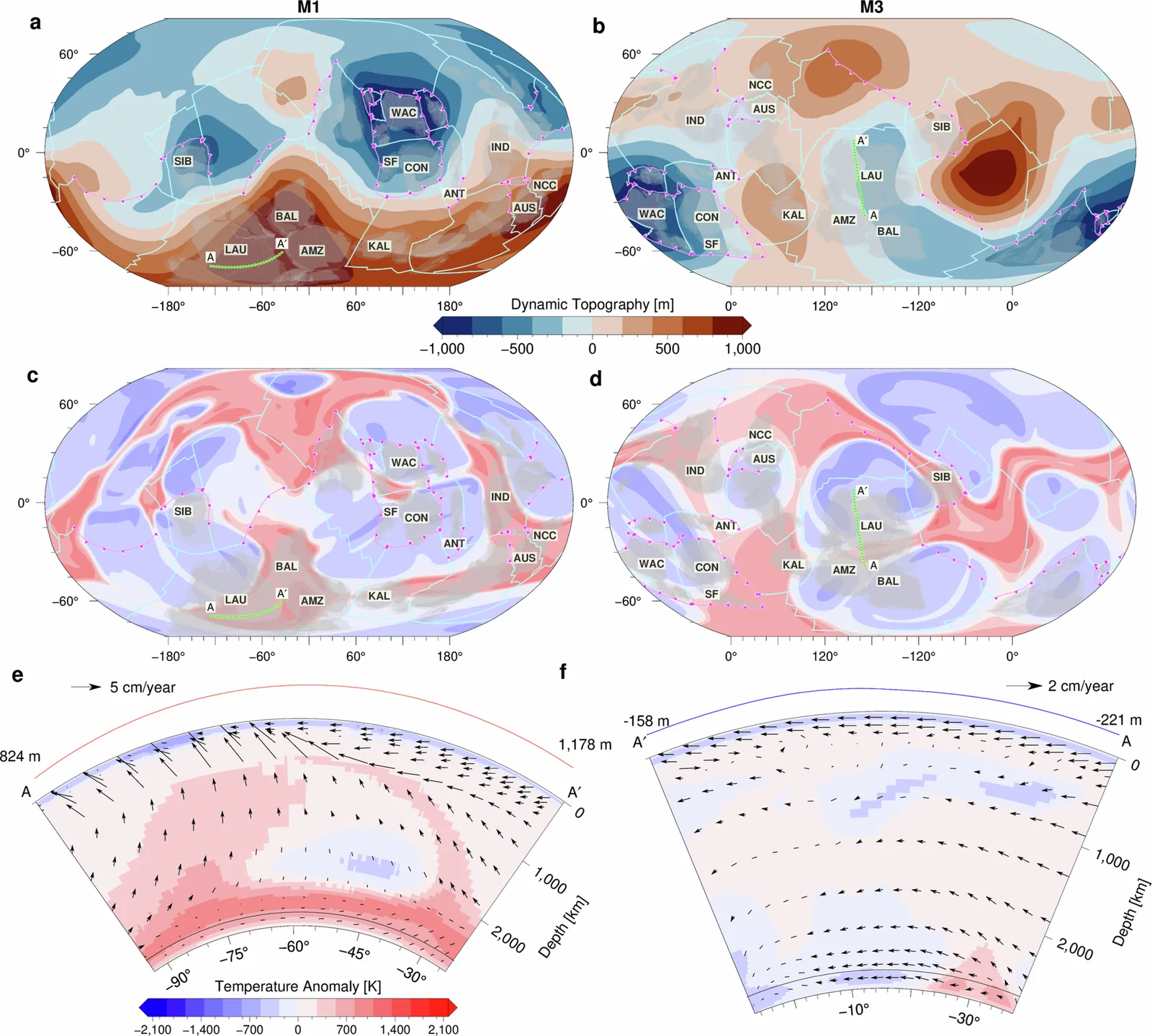
Predicted dynamic topography and mantle temperature at 640 Ma for M1 and M3. **a-b**, Dynamic topography at 640 Ma for M1 (**a**) and M3 (**b**). **c**-**d**, Mantle temperature anomaly at 2,677 km depth at 640 Ma for M1 (**c**) and M3 (**d**). In **a**-**d**, cyan lines represent mid-ocean ridges and transform faults and magenta lines represent subduction zones with triangles on the overriding plate. Transparent grey polygons represent continental blocks, with labels ‘AMZ’: Amazonia, ‘ANT’: Antarctica, ‘AUS’: Australia, ‘BAL’: Baltica, ‘CON’: Congo, KAL: Kalahari, ‘LAU’: Laurentia, ‘NCC’: North China, ‘SF’: São Francisco, ‘SIB’: Siberia and ‘WAC’: West Africa. The green lines with diamonds are great circles transects A-A’ shown in **e**-**f**. The length of the great circle depends on its latitudinal position. Robinson projection at the surface of Earth. **e**-**f**, Cross-sections of temperature anomalies and dynamic topography across Laurentia (transect A-A’) for M1 (**e**) and M3 (**f**). The colour bar in (**e**) is the temperature anomaly scale for **c**-**f**. Black arrows are velocity vectors. The amplitude of dynamic topography (not to scale) is represented above the cross-sections with positive amplitudes in red, negative amplitudes in blue, and reported values at A and A’. The black solid line indicates 2,667 km depth. Polar projection of longitude and depth for M1 (**e**) and of latitude and depth for M3 (**f**).

Importantly, the number of dynamic topography uplift–subsidence cycles governed the formation and preservation of the Great Unconformity. A single cycle of Precambrian uplift followed by Phanerozoic subsidence was the ideal scenario for forming and preserving the Great Unconformity. This scenario, which is in first-order agreement with thermochronology studies^3,4,7,10,18^, was predicted for Laurentia for all models except M3 (Fig. 4a and Extended Data Fig. 8a,f,k,p,v,z). Although Laurentia was in cluster GU-F for model M3 (Extended Data Fig. 2), it was not predicted to have experienced notable uplift and was subsiding above a mantle downwelling at 640 Ma (Extended Data Fig. 9). Together with a poor match of the present-day mantle structure for M3 (Extended Data Fig. 1d), this confirmed that NNR was an appropriate reference frame to model mantle flow^33^, but not PMAG in which lithospheric net rotation was large. Precambrian uplift was predicted for four out of six models for Baltica and North China, with marked variations in amplitude and duration between models (Extended Data Fig. 8b,g,l,q,v,aa), possibly reflecting particularly uncertain subduction history (Supplementary Discussion and Supplementary Table 1). All models predicted dynamic uplift of Baltica as it moved from the southern hemisphere to the northern hemisphere between 540 Ma and 520 Ma (Supplementary Videos 1 and 2), followed by rapid subsidence consistent with a sub-Cambrian unconformity linked to epeirogenic uplift^22,43^.

By contrast, most models predicted two cycles of dynamic uplift followed by dynamic subsidence for South America and Australia and long-lived dynamic subsidence followed by dynamic uplift then renewed dynamic subsidence for Africa (Fig. 4h–j and Extended Data Fig. 8c–ad), consistent with thermochronological and geochronological evidence for these regions^44–48^. The predicted dynamic uplift of South America and Australia coincided with preserved sediment flux on these continents, as expected (Fig. 4h,i and Extended Data Fig. 8c–ac). However, the dynamic uplift of Africa coincided with increased sediment preservation because Africa was positioned centrally in Gondwana (Extended Data Fig. 6b) and orogens in Antarctica and South America (Fig. 3f) restricted drainage from Africa to the oceans, leading to endorheic (inland) sedimentation^49^.

The solution of the presented flow models depends strongly on the tectonic reconstruction, for which the uncertainty is large and increases back in time (Supplementary Discussion and Supplementary Table 1). Testing the sensitivity of the flow model to the tectonic scenario is a challenge that could be addressed by developing alternative tectonic reconstructions with continuously closing plate polygons or by developing a different approach to predict past mantle flow.

## Drivers of the Great Unconformity

Comparing dynamic topography cycles to preserved sediment flux confirmed Laurentia, Baltica and North China as continental blocks on which the Great Unconformity was predicted to have formed and to be preserved (Fig. 4). The Great Unconformity surface is exposed and extensively studied in Laurentia^1,3,5–7,9,10,15,18^ and in North China^8,25–28^, whereas it is mostly inferred to occur beneath younger sediments in Baltica^19–24^. This largely stems from the tectonic and geodynamic evolution of each of these continental blocks. In the considered tectonic scenario, no subduction occurred directly along the margins of Laurentia between about 930 Ma and 510 Ma, Baltica between about 750 Ma and 520 Ma, and North China between about 720 Ma and 540 Ma (Supplementary Discussion, Supplementary Table 1 and Supplementary Videos 1 and 2). Subduction beneath northeast Laurentia from approximately 470 Ma resulted in its amalgamation with Baltica into Laurussia by around 420 Ma, associated with continent-wide Cambrian transgression (Extended Data Fig. 6a and Supplementary Videos 1 and 2). North China was on the periphery of Gondwana during Cambrian times with the Qinling subduction zone system at its western margin from 540 Ma (Fig. 2b, Supplementary Discussion, Supplementary Table 1 and Supplementary Videos 1 and 2). During the assembly of Pangea, collisions occurred along some margins of all three continental blocks (Fig. 3f and Extended Data Fig. 6b), promoting the deformation and erosion of rocks potentially preserving the Great Unconformity, or their burial in foreland basins. The large continental area of Laurentia and limited reworking at its margins were favourable to recording the formation of the continental-scale Great Unconformity. The prominent exposure of the Great Unconformity in the Grand Canyon^15–17^ has been linked to the Laramide uplift event from Farallon flat-slab subduction and subsequent Colorado River incision^50,51^. Repeated orogeny along the eastern margin of Baltica (Fig. 3f) could explain why the Great Unconformity is generally inferred to occur at depth on that smaller continental block. Early Cretaceous lithospheric delamination^52^ could explain the exposure of the Great Unconformity in North China, which is unexpected given its comparatively small area and four episodes of orogenesis between about 500 Ma and 200 Ma (Fig. 3f).

Our models predict changes in dynamic topography that when averaged in time and space by *k*-means clustering occur over approximately 20 Myr and about 5,000 km, suggesting that the motion of continents over the convecting mantle provided the large-scale geodynamic environments that favoured the formation and preservation of the Great Unconformity in Laurentia, Baltica and North China. The geologic record of uplift^4–7,10,53^ and subsidence^4–7,9,10,20^ indicates that the timing of erosion and deposition^54^ varied between locations, which probably reflects the effect of processes, such as tectonic deformation, elastic flexure or shorter wavelength dynamic topography, that would affect relative sea level over shorter spatial and temporal scales.

The amplitude of long-wavelength dynamic topography is thought to be about a tenth of the amplitude of tectonic topography^37^, and continental tilting would not result in the formation of slopes that would lead to sufficient fluvial erosion to explain the magnitude of denudation associated with the Great Unconformity^1,4,7^. We propose that sustained positive dynamic topography throughout Cryogenian times (Fig. 3d) would have resulted in substantial erosion because glacial erosion rates exceed fluvial erosion rates by an order of magnitude and can operate at lower elevations^13^. The rarity of extensive unconformities in the history of Earth could be due to the very long timescale (>900 Myr) of the proposed continental-scale uplift and subsidence to form such an unconformity, combined with rare extensive glaciations and decreasing preservation potential back in geological time.

Our models explain why the Great Unconformity is best preserved in North America and show that it is a first-order surface expression of deep geodynamic processes that transformed global landscapes and sediment fluxes. In turn, these landscape dynamics would have altered seawater chemistry and catalysed the Cambrian explosion of life^2^. Future work could investigate the effects of continental-scale dynamic uplift and subsidence on erosion, sedimentation, seawater chemistry and their consequences for the diversification of life forms.

## Methods

### Macrostrat

Macrostrat is an open, relational geospatial database that enables large-scale, quantitative analysis of 33,903 lithologically and chronologically defined rock units across 1,474 regions in the Americas, New Zealand and the deep sea^55^.

We accessed lithostratigraphic data from the Macrostrat (https://macrostrat.org/api/units/?lith_class=sedimentary&lith_type=metasedimentary) database through theAPI (v.2). We downloaded sedimentary units and associated stratigraphic columns as GeoJSON files in June 2025. We created a map of transgressive sequences and erosional remnants across North America and northern South America (Fig. 1) spanning late Neoproterozoic to early Ordovician times.

### Mantle convection models

We used the code CitcomS^57^ to model mantle convection for a billion years (1,000–0 Ma). In CitcomS, the mantle structure is represented with finite-element nodes in 3D spherical geometry. We considered six model cases (Extended Data Table 1), three of which have been presented in previous work (M4 was MF in ref. ^58^; M5 was C3 in ref. ^59^, C20 in ref. ^33^ and C3 in ref. ^60^; and M6 was C1 in ref. ^59^).

We discretized the mantle into about 13 million nodes (129 × 129 × 65 × 12), with radial mesh refinements at the surface (about 50 × 50 × 15 km) and core–mantle boundary (CMB; about 28 × 28 × 27 km) and relatively lower resolution in the mid-mantle (approximately 40 × 40 × 100 km). We reconstructed mantle flow in a modified version of CitcomS^57,61^ in which plate kinematics and the thermal structure of the oceanic lithosphere and subducting slabs down to 350 km depth were derived from tectonic reconstructions^62^ with continuously closing plate polygons^63^ and imposed as time-dependent boundary conditions in 1 million-year increments. We used the extended Boussinesq approximation that assumed the mantle to be an incompressible fluid to account for viscous dissipation, internal heating, depth-dependent thermal expansion coefficient and an adiabatic temperature gradient^64^.

For initial boundary conditions and time-progressive model simulation, we created a lithospheric thermal structure from reconstructed seafloor ages and half-space cooling with a maximum seafloor age set as 80 Myr to obtain a maximum thickness of oceanic lithosphere consistent with observations for the present day. We used the method in ref. ^65^ to reconstruct synthetic seafloor ages, whereas the continental lithosphere adopted simplified tectonothermal ages from ref. ^66^. The global thermal regime of continental lithosphere and subducting slabs, reconstructed using seafloor ages and location of subduction zones^61^, was imposed in the flow models in increments of 1 million years.

The initial conditions included an adiabatic temperature profile between two boundary layers: the lithosphere at the surface, and a 225 km thick laterally uniform basal layer containing a 113 km thick layer with excess density. The density contrast δ*ρ*_b_ between the basal layer and the ambient mantle was defined by the buoyancy ratio $B=\frac{{\rm{\delta }}{\rho }_{{\rm{b}}}}{\rho \alpha \Delta T}$ where Δ*T* = 4,300 K was the temperature change across mantle for M1–M3 (Δ*T* = 3,600 K for M4 and Δ*T* = 2,800 K for M5 and M6), *ρ* = 5,546 kg m^−3^ was the average density^67^ and *α* = 1.32 × 10^−5^ K^−1^ was thermal expansivity^68^ in the 100 km layer above the CMB. The initial condition consisted of slabs inserted down to 1,000 km depth for models M1–M4 (1,200 km for models M5 and 550 km for M6) with dip angles of 45° dip down to 425 km depth and 90° descent below. To incorporate the advective thickening of more viscous lower mantle, lower mantle slabs were twice as thick as upper mantle slabs in the initial condition. Models were initialized with differing warm-up phases lasting 250 Myr for models M1 and M4, 200 Myr for models M6 and 0 Myr for other models (Extended Data Table 1) using the 1 Ga configuration for tectonic velocities, subduction zone positions and lithospheric age. Boundary conditions were isothermal at the surface and at the CMB, imposed plate kinematics (velocities) at the surface and a free-slip kinematic condition at the CMB.

The convective vigour in models was governed by the Rayleigh number ${\rm{R}}{\rm{a}}=\frac{{\alpha }_{0}\rho g\Delta T{h}_{{\rm{M}}}^{3}}{\kappa {\eta }_{0}}$, where *α*_0_ = 3 × 10^−5^ K^−1^ was the coefficient of thermal expansion at the surface, *ρ* = 4,000 kg m^−3^ was the density, *g* = 9.81 m s^−2^ was the gravitational acceleration, *h*_M_ = 2,867 km was the thickness of the mantle, *κ* = 1 × 10^−6^ m^2^ s^−1^ was the thermal diffusivity, *η*_0_ = 1 × 10^21^ Pa s was the reference viscosity. The subscript ‘0’ indicated reference values. The coefficient of thermal expansion was depth-dependent, decreasing by a factor of about 2 over the thickness of the mantle (see refs. ^68,69^). Based on these values, we obtained Ra = 1.2 × 10^8^ for M1–M3. The Rayleigh number was varied across model cases (Extended Data Table 1).

Likewise, the dissipation number governed heat dissipation generated by shear heating. It was given by ${\rm{D}}{\rm{i}}=\frac{{\alpha }_{0}g{h}_{{\rm{M}}}}{{C}_{p}}$, where 2867 *C*_p_ = 1,200 J kg^−1^ K^−1^ was the heat capacity. The total rate of internal heating was equal to 33.6 TW, accounting for contributions from both radioactive decay (about 9–17 TW; ref. ^70^) and primordial heat dissipated through secular cooling of the interior of Earth (around 8–29 TW; ref. ^70^).

Depth-, composition-, temperature- and pressure-dependent viscosity was computed as

1 $$\eta =\eta (r)\,{\eta }_{{\rm{C}}}\exp \left\{\frac{{E}_{\eta }+\rho g{Z}_{\eta }(R-r)}{{R}_{{\rm{u}}}(T+{T}_{{\rm{o}}{\rm{f}}{\rm{f}}})}-\frac{{E}_{\eta }+{\rho gZ}_{\eta }(R-{R}_{{\rm{c}}})}{{R}_{{\rm{u}}}({T}_{\mathrm{CMB}}+{T}_{{\rm{o}}{\rm{f}}{\rm{f}}})}\right\}.$$

The depth-dependent viscosity pre-factor *η*(*r*) was equal to 2 × 10^19^ Pa s above 160 km depth and between 310 km and 660 km depth (upper mantle), to 6 × 10^17^ Pa s in the asthenosphere between 60 km and 310 km depth for models M1–M3 (2 × 10^18^ Pa s for models M4–M6), and 6 × 10^20^ Pa s in the lower mantle below 660 km depth for models M1–M3 (2 × 10^20^ Pa s for models M4–M6; Extended Data Table 1). The compositional viscosity pre-factors *η*_C_ were equal to 1 for ambient mantle, to 100 for continental lithosphere (modelled as in ref. ^66^), and to 10 for the basal layer. The term *r* was the radius, *R*_c_ = 3,504 km was the radius of the core, *E*_*η*_ = 284 kJ mol^−1^ was the activation energy, *Z*_*η*_ = 2.1 × 10^−6^ m^3^ mol^−1^ was the activation volume, *R*_u_ = 8.31 J mol^−1^ K^−1^ was the universal gas constant, *T* was the dimensional temperature, *T*_off_ = 694 K was a temperature offset and *T*_CMB_ = 4,300 K was the temperature at the core–mantle boundary. The viscosity structure, defined by the viscosity pre-factors, activation volume, activation energy and temperature offset produced viscosity variations varying over three orders of magnitude across the model^33^. Although these contrasts were smaller than expected for the solid Earth^71^, these lateral viscosity variations could be resolved at a spatial resolution that allows time-dependent mantle flow to be computed using up to about 15,000 CPU hours per 100 million years on supercomputer Gadi.

We derived boundary conditions from the global tectonic reconstructions in ref. ^62^ (which merges with tectonic reconstructions in refs. ^72–75^), which was the only tectonic reconstruction extending back to 1 billion years ago with continuously closing plate polygons that are necessary in models based on the approach in ref. ^61^. We considered two reference frames for the tectonic reconstruction in ref. ^62^: the original palaeomagnetic reference frame (PMAG, model M3) and a no-net-rotation reference frame (NNR), which was suitable to model mantle convection^33,76^. We analysed model results, including dynamic topography, mantle temperature and composition fields in increments of 20 million years.

We computed air-loaded dynamic topography at the surface of Earth as $h=\frac{{\sigma }_{\mathrm{rr}}}{\Delta \rho g}$, where *g* = 9.81 m s^−2^ was the acceleration of gravity and Δ*ρ* was the difference in densities between air (*ρ*_A_ = 0 kg m^−3^) and the shallow upper mantle (*ρ*_UM_ = 3,320 kg m^−3^). We considered free-slip boundary conditions, contributions from sources of buoyancy deeper than 350 km depth, and lateral viscosity variations deeper than 350 km depth.

### Evaluating model success

As in refs. ^32,33,77^, we first classified the present-day lower mantle structure as mapped by 10 independent S-wave tomographic models^78–87^ and predicted by six mantle flow models (M1–M6). To achieve this, we generated 196,965 evenly spaced points (about 0.45°) on the surface and sampled mantle temperature (seismic velocity) approximately every 58 km between 1,000 km and 2,800 km depths. We then used *k*-means^88^ clustering to group these points into user-specified *k* clusters such that the within-cluster sum of squares (WCSS), that is, the squared Euclidean distances between points and the centroid of the group was minimized.

As in previous studies^32,33^, we examined model success by computing three metrics (Extended Data Fig. 1): (1) the predicted fractional area *f*_a_ of the surface of Earth covered by clusters for hot BLOBSs (for mantle flow models) and slow LLSVPs (for tomographic models), (2) the global accuracy ($\overline{\mathrm{Acc}}$), which was a measure of the spatial match between the present-day mantle structure (clusters) predicted by mantle convection models and that averaged across 10 independent tomographic models given by $\overline{\mathrm{Acc}}=\frac{\mathrm{TP}+\mathrm{TN}}{A}$, where TP was the area of true positives representing spatial match between hot BLOBSs and LLSVPs, TN the area of the true negatives measuring the spatial extent of cold basal mantle structure and high seismic velocity profiles and *A* was the total surface area of Earth. For each model, the mean accuracy $\overline{\mathrm{Acc}}$ represented the average across 10 tomographic models, whereas for each tomographic model, $\overline{\mathrm{Acc}}$ was computed as an average across the nine independent tomographic models; and (3) the time-averaged median angular distance $\widetilde{\theta }$ between reconstructed volcanic eruption locations (LIPs and kimberlites as in ref. ^33^) from 320 Ma and the edge of hot BLOBSs. The distance was taken as zero for eruption locations above a BLOBS or LLSVP. Smaller distances indicated a close spatial match between BLOBSs or LLSVPs and volcanic eruptions, which was thought to be favourable^32,33,89^. Mantle flow models were deemed to perform better if all three metrics were within the range or close to the range for tomographic models (Extended Data Fig. 1, grey areas).

### Cluster analysis of dynamic topography

We generated a set of evenly spaced (about 0.45°) points entirely covering present-day continents. We extracted dynamic topography at the reconstructed location of these points in 20 Myr intervals and rotated the results back into the present-day plate configuration. We used the continental blocks that continuously existed from 920 Ma to mask data for all other times so that the considered locations existed for all considered times. We used *k*-means clustering^88^ as in ref. ^90^ to classify geographic regions that share a similar evolution of predicted dynamic topography.

We used the silhouette^91^ score, Davies–Bouldin index^92^ (DBI) and elbow method^93^to determine the appropriate number of clusters to consider (Supplementary Fig. 1). The silhouette score measured how similar a point was to its own cluster compared with other clusters. It combined intra-cluster cohesion and inter-cluster separation. Silhouette scores ranged from −1 to 1, with larger values indicating better-defined clusters. The DBI evaluated intra-cluster similarity and inter-cluster differences based on intra-cluster dispersion and inter-cluster distance. Lower scores indicated better clustering. It was computationally efficient but assumed compact, spherical clusters, making it less reliable for irregular shapes. The heuristic elbow method consisted of plotting the number of clusters (*k*) against the clustering cost (for example, WCSS) and identifying the ‘elbow’ point, at which the rate of improvement (decrease in WCSS) decreased sharply, suggesting the most appropriate *k*.

We identified the ideal number of clusters for all six model cases for both the Great Unconformity formation (920–420 Ma) and preservation (420–0 Ma) periods to be equal to either two or three (Supplementary Fig. 1). We produced cluster maps for each model case and each cluster combination (either two or three) and combined them in maps indicating model agreement with a score between zero and six out of six models. These ‘vote’ maps highlighted regions showing both formation and preservation of the Great Unconformity across model and cluster combinations (Extended Data Fig. 5).

### Mapping temporal evolution of preserved sediment flux and dynamic topography

We obtained the preserved sediment flux (sediment volume divided by time) from Neoproterozoic times (1.6 Ga) to the present for North America, South America, Africa, Eurasia and Australia from ref. ^1^. For the period 920–0 Ma, we scaled sediment flux by the present-day area of each considered continent as in ref. ^1^. We averaged continental dynamic topography for each continental block that continuously existed between 920 Ma and 0 Ma (Extended Data Fig. 8a) using the time periods at which sediment volume data were defined (Fig. 4a and Extended Data Figs. 7 and 8).

To focus on long-term, long-wavelength processes, we filtered the preserved sediment data using time windows that corresponded to the periods of dynamic uplift and dynamic subsidence predicted for each continent. We then averaged the preserved sediment volume to represent the sediment accumulation associated with each phase of dynamic topography (Fig. 4f–j).

### Mapping subduction convergence rate

The subduction convergence rates at trench segments were computed using plate-kinematic workflow of GPlately (v.2.0.0), built atop PyGPlates^94^, a Python interface for GPlates^95^ and PlateTectonicTools. The process consisted of resolving the topological plate model and extracting subduction zones from the network of dynamic plate boundaries reconstructed at a specified age using the NNR model. Each subduction zone was then tessellated, that is, discretized into a series of evenly spaced points using the ‘tessellation_subduction_zone’ module in GPlately. At each tessellated point, subduction convergence rate, obliquity and trench migration rate were calculated from the relative motion of the down-going and overriding plates at each reconstruction time step. Negative convergence-rate values, which indicated infrequent inconsistencies in the model with divergence occurring at a convergent boundary^72^, were set to zero before computing the range of convergence rates for each subduction zone listed in Supplementary Table 1.

## Online content

Any methods, additional references, Nature Portfolio reporting summaries, source data, extended data, supplementary information, acknowledgements, peer review information; details of author contributions and competing interests; and statements of data and code availability are available at https://doi.org/10.1038/s41586-026-11156-5.

## Supplementary information

Supplementary Information This file contains Supplementary Discussion, Supplementary Table 1, Supplementary Fig. 1, legends for Supplementary Videos 1 and 2, and Supplementary References. Supplementary Discussion: Discussion of the key subduction zones driving the predicted pattern of dynamic topography between 740 Ma and 440 Ma. Supplementary Table 1: Characteristics and geological evidence for the major subduction zones inferred to have driven mantle flow between 740 Ma and 440 Ma. Supplementary Fig. 1: Statistical determination of the number of clusters based on the silhouette score, Davies–Bouldin index (DBI) and elbow method.

Peer Review File

Supplementary Video 1 Animation showing the predicted joint evolution of dynamic topography and mantle temperature from 920 Ma. See Supplementary Information for full legend.

Supplementary Video 2 Animation showing the predicted evolution of dynamic topography and input subduction convergence rates for each subduction zone between 740 Ma and 440 Ma. See Supplementary Information for full legend.

## Figures and Tables

**Extended Data Table 1:**
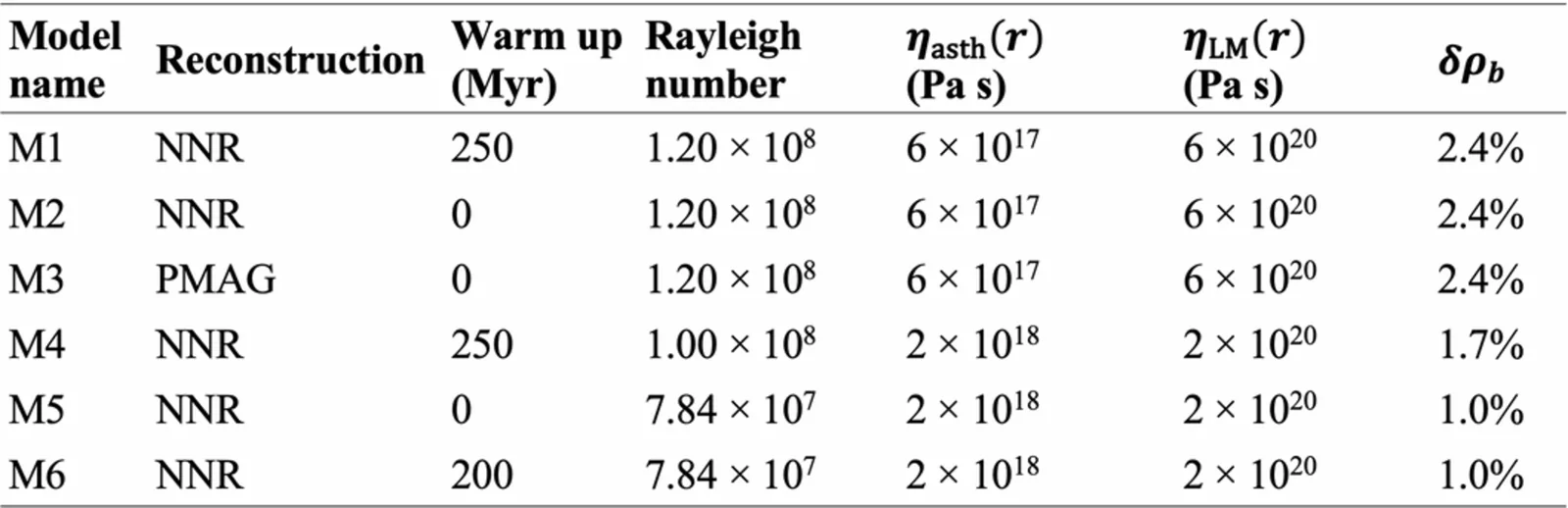
Input parameters and boundary conditions for mantle flow model cases

## Data Availability

Data generated for this study can be accessed at Code Ocean (https://doi.org/10.24433/CO.2189114.v1) and Zenodo^96^ (https://doi.org/10.5281/zenodo.17773493).

## Code availability

The code used to run mantle models is available at GitHub (https://github.com/EarthByte/citcoms). The Jupyter notebooks and Python scripts written to process data and generate Figs. 1–4, Extended Data Figs. 1–9 and Supplementary Fig. 1 are available at Zenodo^96^ (https://doi.org/10.5281/zenodo.17773493) and in Code Ocean capsule (https://doi.org/10.24433/CO.2189114.v1). Figures 1–3 and Extended Data Figs. 2–6 and 9 were created using the open-source Python library PyGMT (v.0.13.0), a Pythonic interface for the Generic Mapping Tools (GMT)^97^, and Fig. 4, Extended Data Figs. 1, 7 and 8 and Supplementary Fig. 1 were created using the open-source Python library Matplotlib^98^ (v.3.8.3).
