# Supplementary material for: The deep Earth origin of the Great Unconformity
Source: Nature. 2026 Nov 4;659(8139):106–12. doi: 10.1038/s41586-026-11156-5 (PMC13736641; doi:10.1038/s41586-026-11156-5)
Supplement: Supplementary file 1 [file 41586_2026_11156_MOESM1_ESM.pdf]

nature portfolio

https://doi.org/10.1038/s41586-026-11156-5

Supplementary information

The deep Earth origin of the Great Unconformity

In the format provided by the authors and unedited

Supplementary Information for:
The Deep Earth Origin of the Great Unconformity

Santosh Dhungana[1], Nicolas Flament[1*]

[1]Environmental Futures, School of Science, University of Wollongong, Northfields Avenue, NSW 2522, Australia

1. Supplementary Discussion

The subduction zones mentioned in the text below are listed in Supplementary Table 1 and shown in Supplementary Video 2. References have been made to articles introducing hemispheric tectonic reconstructions[73,74] and global tectonic reconstructions with continuously closing plates[62,75], in which the timing and extent of subduction zones are approximative as it impractical to capture details of regional geology at that scale. The reader is referred to these four references, and references therein, for further details.

Positive dynamic topography due to limited subduction along the margins of Laurentia, Baltica and North China during Cryogenian times (720–640 Ma)

Laurentia

A key aspect of the tectonic reconstruction of ref. 62 – M21 – is the absence of subduction zones along the margins of Laurentia between 930 Ma and 510 Ma, when the well-established Lushs Bight subduction zone (LBS) started to its east[73] (here all orientations are given with respect to continental blocks reconstructed in the no-net-rotation frame of reference). In our mantle flow models, subduction to the northeast of Laurentia (LSZ) with a large convergence rate was key in driving its accelerated subsidence from 470 Ma. This subduction zone, which resulted in the formation of Laurussia through the Caledonian orogeny, is well-established from the geology of Northeast Greenland and Svalbard[73].

In M21, a hypothetical Missing Link Ocean separated Laurentia from Australia and North China between 700 Ma and 635 Ma, which was bordered to its north by the south-dipping Missing Link subduction zone (MLS). A speculative intra-oceanic subduction zone (IOS) to the west of Laurentia, separated from the continental block by a mid-ocean ridge, was used to accommodate the well-established rifting and latitudinal motion of Laurentia between 590 Ma and 540 Ma[62]. Downwelling associated with these two subduction zones with intermediate to fast convergence rates restricted the western extent of the dynamic upwelling beneath Laurentia between 700 Ma and 540 Ma.

Baltica

In M21, there is limited subduction along the margins of Baltica between 750 Ma and ~520 Ma, with the speculative Timan intra-oceanic subduction (TiSZ) resulting in some dynamic subsidence from ~590 Ma. The motion of Baltica towards the Taimyr subduction zone (TaSZ) caused dynamic subsidence down to the west from ~560 Ma[62], followed by dynamic subsidence down to the east as Baltica moved close to Avalonia by 520 Ma[73]. In M21, this motion is accommodated by the Cadomian subduction zone (ACS) until 520 Ma, followed by a speculative intra-oceanic subduction zone (OBS) until 460 Ma to accommodate plate motion inferred from paleomagnetic data[62].

North China

In M21, North China was not in the immediate vicinity of a subduction zone between 720 Ma and 540 Ma. Peri-Australia subduction (PAS) to the northwest of North China between 750 Ma and 720 Ma is speculative, kinematically required based on the proposed locations of Australia, Lhasa and North China. Subduction around the adjacent Lhasa block (Lhasa-Australia convergence, LAC, and Lhasa-India convergence, LIC) between 650 Ma and 600 Ma is also speculative and kinematically required; the convergence rate is small there and so is the effect on dynamic topography.

The large uncertainty about the subduction zones surrounding Baltica and North China between 750 Ma and 460 Ma could explain why our models do not consistently predict the formation of the Great Unconformity in Baltica and North China, in contrast to Laurentia (Extended Data Figures 4 and 5).

Main subduction zones driving negative dynamic topography during Cryogenian times (720–640 Ma)

Sahara Metacraton and Congo Craton

The two main areas of dynamic topography at 640 Ma are associated with subduction at the margins of African and Siberian continental blocks. Subduction along the margins of the Sahara Metacraton from 750 Ma is inferred from limited outcrop data in a desertic region, with the largest convergence rate to the north of the Sahara Metacraton. While the details of the complex subduction network are uncertain, the rock record indicates that subduction is likely to have occurred in the area between 750 Ma and 640 Ma[62]. Subduction along Mozambique (MZB) from 700 Ma is kinematically inferred to close the Mozambique Ocean and form the Mozambique Belt, and well-established subduction along the Congo from 650 Ma (ZLD) is tentatively proposed to have extended offshore, linking to subduction around the São Francisco craton where the Brasilia Belt (BRB) formed[62].

Siberia

Subduction along the margins of Siberia included the well-established long-lived Taimyr subduction zone (TaSZ) with Siberia as the overriding plate until 700 Ma, then as the subducting plate, and subduction associated with the Isakov Island arc (ISA) from 700 Ma (ref. 62).

Unconstrained longitudinal position

The past longitudinal position of continents is unknown, and the limited longitudinal motion of the Saharan Metacraton, Congo Craton and Siberian Craton between 740 Ma and 640 Ma in M21 contributes to the establishment of well-developed negative dynamic topography by 640 Ma.

Key subduction zones driving the dynamic subsidence of Laurentia, Baltica and North China during Cambrian and Ordovician times (540–440 Ma)

Laurentia

As mentioned above, subduction to the northeast of Laurentia (LSZ) was key in driving its accelerated subsidence from 470 Ma. In M21, subduction along its eastern margin was established by 510 Ma and persisted until 380 Ma. The closure of the Iapetus Ocean by the east-dipping Popelogan–Victoria subduction zone (PVS) and the west-dipping Dashwoods terrane subduction zone (DWT) was followed by the accretion of Avalonia and Ganderia to Laurentia by subduction zones WAS and GSZ[62,73].

Baltica

In addition to the above-mentioned Offshore Baltica subduction zone (OBS), the speculative fast-converging, short-lived North Kara subduction zone (NKS) and the proposed extension of East Avalonia subduction (EAS) to the margin of Baltica from 440 Ma contribute to the modelled dynamic subsidence of Baltica during Cambrian and Ordovician times. We reiterate that the subduction history at the margins of Baltica between 750 Ma and 440 Ma is particularly uncertain in M21.

North China

The early Paleozoic dynamic subsidence of North China was driven by the Bainaimiao-Ondor Sum subduction zone (BOS) from ~490 Ma and the Qinling subduction zones (QIS and NQB) from ~510 Ma[74].

2. Supplementary Table 1 | Characteristics and geological evidence for the major subduction zones in the tectonic reconstruction by ref.62 inferred to have driven mantle flow between 740 Ma and 440 Ma.

Subduction zone name (abbreviation)	Age (Ma)	Dip direction	Convergence rate (cm/yr)	Evidence	Refs
Surrounding Laurentia					
Missing Link (MLS)	700–635	South	4.69–10.30	None; kinematically required	62
Intra-oceanic (IOS)	590–540	East	3.31–18.12	None; kinematically required	62
Lushs Bight (LBS)	510–490	West	0.00–4.04	Accreted arc	73
Dashwoods terrane (DWT)	490–465	West	0.00–3.02	Accreted terrane	73
Popelogan-Victoria (PVS)	480–440	East	4.66–16.00	Accreted arc	73
Laurentian (LSZ)	470–430	SW	5.21–23.27	Accreted terrane	62,73
Ganderia (GSZ)	440–430	West	0.00–16.28	Accreted terrane	62,73
West Avalonia (WAS)	440–420	West	0.00–3.02	Accreted terrane	62,73
Surrounding Baltica					
Timan (TiSZ)	700–585	E then W	8.42–11.25	None: unidentified Timan arc	75
Offshore Baltica (OBS)	520–475	East	2.39–14.20	None; kinematically required	62
North Kara (NKS)	470–450	East	24.46–42.62	None; kinematically required	62
East Avalonia (EAS)	440–420	West	0.00	Accreted terrane	62,73
Surrounding North China					
Peri-Australia (PAS)	750–720	SE	0.00–6.48	None; kinematically required	62
Lhasa-Australia (LAC)	650–600	South	0.00–2.85	None; kinematically required	62
Lhasa-India (LIC)	650–600	North	0.00–2.85	None; kinematically required	62
Bainaimiao-Ondor Sum (BOS)	490–430	West	0.00–2.61	Bainaimiao arc and Ondor Sum complex	74
Qinling (QIS)	540–440	E then W then both	0.13–4.71	Subduction-related magmatic rocks and (U)HP	74
North Qinling (NQB)	440–420	East	0.00–24.47	Subduction-related magmatic rocks and (U)HP	74
Surrounding the Sahara Metacraton, Congo Craton and São Francisco Craton					
Subduction network around Sahara Metacraton	750–640	E, W and S	0.00–15.53	Accreted terranes (limited outcrop evidence)	75
Mozambique Belt (MZB)	700–600	West	0.00–1.36	Orogen (metamorphic rocks)	75
Zambezi-Lufilian-Damara Belt (ZLD)	650–550	North	0.00–5.26	Orogen (metamorphic rocks); speculative before 600 Ma	75
Brasilia Belt (BRB)	650–615	East	0.00–4.64	Accreted terranes; uncertain timing	75
Avalonia-Cadomia (ACS)	650–520	East	0.00–11.10	Accreted terrane, volcanic arc	62,75
Surrounding Siberia					
Taimyr (TaSZ)	700–601	North	0.40–0.54	Accreted terrane	75
Isakov Arc (ISA)	650–615	East	0.00–3.81	Accreted arc	62

Dip directions are given with respect to continental blocks reconstructed in the no-net-rotation frame of reference. Abbreviations are for cardinal directions.

Ranges are provided for the age and the convergence rate.

3. Supplementary Video 1 | Predicted joint evolution of dynamic topography and mantle temperature from 920 Ma. a, Dynamic topography. **b**, Mantle temperature anomaly at 2,677 km depth. In **a**-**b**, cyan lines represent mid-ocean ridges and transform faults, and magenta lines represent subduction zones with triangles on the overriding plate. The green lines with diamonds are great circles transects A-A' shown in **e**-**f**. The length of the great circle depends on its latitudinal position. Robinson projection at Earth's surface. Transparent grey polygons represent continental blocks, with labels 'AMZ': Amazonia, 'ANT': Antarctica, 'AUS': Australia, 'BAL': Baltica, 'CON': Congo, KAL: Kalahari, 'LAU': Laurentia, 'NCC': North China, 'SF': São Francisco, 'SIB': Siberia and 'WAC': West Africa. **c**, Cross-section of temperature anomalies and dynamic topography across Laurentia (transect A-A'). The dynamic topography line (not to scale) above the cross-section plot illustrates spatial variations, with positive values shown in red and negative values in blue. The maximum and minimum values of dynamic topography along the transect are reported at their respective locations above the line. The black solid line indicates 2,667 km depth. Polar projection of longitude and depth. The colour bar in (**c**) is the temperature anomaly scale for **b**-**c.**

4. Supplementary Video 2 | Predicted evolution of dynamic topography and input subduction convergence rates between 740 Ma and 440 Ma. a, Dynamic topography**. b,** Convergence rate along the subduction zones. In **a (b)**, cyan (red) lines represent mid-ocean ridges and transform faults, and magenta (black) lines represent subduction zones with triangles on the overriding plate. Robinson projection at Earth's surface. Translucent grey polygons represent continental blocks and label abbreviations are 'BAL': Baltica, 'CON': Congo, 'LAU': Laurentia, 'NCC': North China, 'SMC': Sahara Metacraton and 'SF': São Francisco. Subduction zones mentioned in Supplementary Table 1 are labelled. Grey arrows are plate velocity vectors.

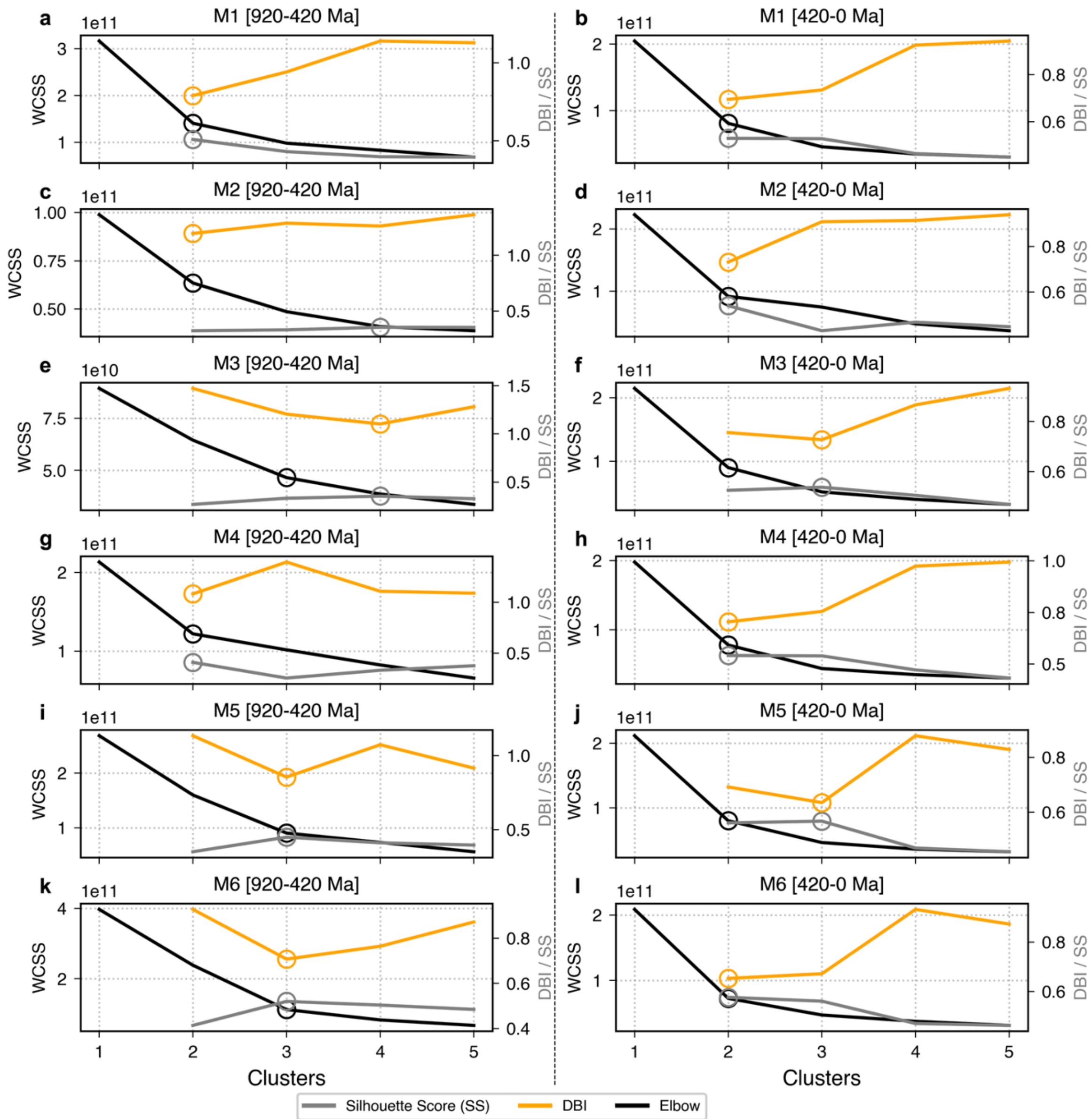

5. Supplementary Figure 1 | Statistical determination of the number of clusters based on the Silhouette score, Davies-Bouldin Index (DBI) and elbow methods. a-b, Scores for each metric against the number of clusters for the data set corresponding to 920–420 Ma (**a**) and 420–0 Ma (**b**). Coloured rounded markers in **a-b** indicate the optimal number of clusters based on the score of each metric (grey: Silhouette score, orange: DBI and blue: elbow method). Maximum score, minimum score and bends in plot are the criteria used by Silhouette, DBI and elbow method respectively to determine optimal number of clusters. M1-M6 denote the six considered model cases. 'WCSS' is the within-cluster sum of squares. Cartesian projection in (**a-b**).

References (numbered as in the Methods section)

62 Merdith, A. S. et al. Extending full-plate tectonic models into deep time: Linking the Neoproterozoic and the Phanerozoic. *Earth-Science Reviews* **214**, 103477 (2021). https://doi.org/10.1016/j.earscirev.2020.103477

73 Domeier, M. A plate tectonic scenario for the Iapetus and Rheic oceans. *Gondwana Research* **36**, 275-295 (2016). https://doi.org/10.1016/j.gr.2015.08.003

74 Domeier, M. Early Paleozoic tectonics of Asia: Towards a full-plate model. *Geoscience Frontiers* **9**, 789-862 (2018). https://doi.org/10.1016/j.gsf.2017.11.012

75 Merdith, A. S. et al. A full-plate global reconstruction of the Neoproterozoic. *Gondwana Research* **50**, 84-134 (2017). https://doi.org/10.1016/j.gr.2017.04.001
