# Supplementary material for: The deep Earth origin of the Great Unconformity
Source: Nature. 2026 Nov 4;659(8139):106–12. doi: 10.1038/s41586-026-11156-5 (PMC13736641; doi:10.1038/s41586-026-11156-5)
Supplement: Supplementary file 2 [file 41586_2026_11156_MOESM2_ESM.pdf]

nature portfolio

Peer Review File

The Deep Earth Origin of the Great Unconformity

Corresponding Author: Dr Nicolas Flament

Any redactions in this file are there to maintain patient confidentiality, the confidentiality of unpublished data, or to remove third-party material.

This file contains all reviewer reports in order by version, followed by all author rebuttals in order by version.

Version 0:

Reviewer comments:

Referee #1

(Remarks to the Author)
Review of "The Deep Earth Origin of the Great Unconformity" by S. Dhungana and N. Flament

This manuscript reports a very interesting finding linking the Great Unconformity to the large-scale, long-term evolution of the Earth's mantle and the resulting dynamic topography. Using a mantle flow model driven by plate motions and boundaries derived from a deep-time plate reconstruction model, the authors show that only a few areas of the present-day continental surface present the uplift/subsidence history (caused by dynamic topography) conducive to the formation and preservation of the Great Unconformity. Interestingly, these areas coincide with the present locations where this feature has been documented. Furthermore, the authors also show that their model predicts erosion/sedimentation patterns that are (for the most part) consistent with a global atlas of preserved sediment flux.

1. General assessment

The argument developed by the authors is very compelling. It starts with a plate reconstruction model that spans 1 Byr of Earth's history that is used to drive a global mantle flow model, which, in turn, is used to compute dynamic topography. A cluster analysis is then used to show that only a few continental areas have experienced the optimum dynamic topography scenario to produce an unconformity between 920-420 Ma and preserve it afterwards. The authors show that these areas coincide with those where the Great Unconformity is best observed/preserved, i.e., Western Laurentia, Baltica, North China, and Siberia.

I read this manuscript with great enthusiasm and believe it contains material that definitely deserves to be published. The authors took great care in assessing the sensitivity of the results to the model parameters (initial condition, viscosity, and density structure) and used a robust and objective means to compare the predictions to observations (the cluster analysis).

There is no doubt that the material presented in this manuscript is of interest to Nature readership. It is likely to have a very large impact. And I therefore recommend its publication.

2. Concern

However, I have an important concern about the findings: from what is presented and explained in the manuscript (the manuscript itself, the method section, and the extended data section), it is clear that the patterns of the mantle flow (and thus the patterns and history of dynamic topography) are entirely driven by the assumed plate reconstruction over the last 1 Byr. Unfortunately, the sensitivity of the model results to the assumed plate reconstruction has not been assessed.

In the mantle flow model, surface velocities are imposed, as well as the position of plate boundaries, and the flow induced by subducting slabs. This explains why the results (dynamic topography predictions) are only mildly dependent on the viscosity/density structure and initial conditions. For most, the flow is not dynamical (i.e., driven by internal density variations) but a direct consequence of the imposed boundary conditions. We can see from the model results (Figure 2) and as explained in the text, that positive dynamic topography arises above a BLOB and negative dynamic topography arises in regions surrounded by subduction zones. The BLOB is, in turn, generated as a return flow to the imposed subducting slabs.

This indicates that the solution of the flow model is strongly dependent on the assumed plate reconstruction, i.e. the location of subduction zones that strongly affects the position of subducting slabs that are the main drivers of the flow and the location of mid-ocean ridges (MORs) that fully controls the thermal structure of the oceanic lithosphere. There exist, however, many alternate plate reconstruction scenarios that could/should be tested. Showing how the results depend on the assumed plate reconstruction would offer a much more convincing argument that the Great Unconformity is indeed produced by dynamic topography. From what is presented here and in the manuscript describing the plate reconstruction model (Merdith et al., 2021), it is very difficult to assess its uncertainty. Merdith et al. (2021) themselves state: "This study is intended as a first step in the direction of a detailed and self-consistent tectonic reconstruction for the last billion years of Earth history". I am, in particular, worried about the uncertainty on the location of MORs and, to a degree, of the subduction zones.

I realize that a large amount of work and computations were needed to arrive at the results presented in this manuscript. But could the authors perform similar computations with different plate reconstructions or with a similar plate reconstruction but "perturbed" within the uncertainty of the geological constraints? If this is not feasible, and in view of the fact that the pattern of dynamic topography can be relatively easily "guessed" from the geometry of the plate boundaries (see my comment above), the authors should be able to assess and present whether alternate plate reconstruction models are likely (or not) to produce similar patterns of dynamic topography. This would be a very worthwhile and instructive exercise that would strengthen the authors' findings (or not).

I believe such a "test" is needed, especially in view of the authors' concluding remark that: "A new opportunity is opening in Earth system modelling to unravel the geodynamic environments of the evolution of complex life by coupling billion-year reconstructions of Earth's evolving deep interior and landscapes to the evolution of global sediment fluxes, sea water chemistry, and diversification of life forms." If indeed, one should consider the reconstructed mantle flow presented here as a basis for further studies, it needs (a) to be better stated that the model is strongly controlled by the assumed plate reconstruction model and (b) to be shown how sensitive the mantle flow model is to that particular plate reconstruction model.

3. Suggestion

I note that the discussion (rightly) focuses on how the model explains the conditions for the formation of the Great Unconformity (its extent, its timing, and preservation). It would also be very interesting to understand why such a feature is relatively rare in Earth's history. Is it due to a peculiar distribution of continental masses above the convective mantle? Or is it a consequence of an evolution of convective flow regime at the end of the so-called "boring billion" (period of reduced plate tectonic activity)? Or is it a question of preservation? I believe the authors have all the elements necessary to add a short paragraph in their discussion section that would give the manuscript a greater impact.

I hope the authors will find these comments/suggestions constructive.

4. Readability and minor points

I found this manuscript relatively well written and fairly pleasant to read. However, a few points need to be clarified or improved as discussed in some of the minor points below. I also note that to fully understand the methodology used, and its implications on the findings, requires a thorough inspection of the method section, the extended data section, and several key references. Although the findings reported here have great potential to be published in a broad journal like Nature, this manuscript could really benefit from a longer format.

4.1: Lines 20-22: This sentence is a repetition of lines 15-18.

4.2: Line 34. It is not clear where the number 1200 Myr comes from and what it refers to: is it the maximum extent of the Great Unconformity at a given place on Earth? It seems unlikely that a single process would be responsible for an unconformity that would last this long.

4.3 Figure 1: As far as I can tell, this figure is not referred to in the text of the manuscript. It is not clear what it represents as an unconformity is defined as a feature that corresponds to a lack of sedimentation (or removal of sediment). So to what correspond the "oldest sedimentary deposits of the Great Unconformity": are they at the base of the unconformity (or the oldest sediment on top of it) or do they correspond to sediments deposited during the great unconformity (in which case they show the locations of where the unconformity is not)? In any case, I do not think that this is a very good "opening" figure for the article. I would try to have a more direct expression of what is and where is observed the Great Unconformity.

4.4 Line 79: That buoyancy and viscosity variations deeper than 350 km depth only are considered here implies that any source of dynamic topography nearer the surface is neglected, including any "blobbing" or "delamination" of parts of the lithosphere. This should be made clearer here. There are many examples of long-lasting, relatively large features such as the subsidence of the Congo Basin that have been explained with a near-surface source (Downey and Gurnis, 2009, for example).

4.5 Figure 2: It is unfortunate that the label for Laurentia (LAU) is partly obscured by other labels in several of the panels. Please correct as Laurentia is a key element of this figure.

4.6 Figure 3: Panel f is very small. I am not sure how useful it will be once (potentially) reduced to publication size.

4.7: Lines 198-201. Interestingly, there is also evidence for strong subsidence and uplift of the Slave Craton during the Phanerozoic (Ault et al., Tectonics, 2013), which contradicts this statement.

4.8 Lines 464-466: I do not think that the term "assimilated" is correct here. The model is driven by plate kinematics, plate boundaries, and the assumed positions of subduction zones and MORs. As stated in Bowers et al. (2015): "The methods consist of four constraints determined a priori from a plate history model: (1) plate velocities, (2) thermal structure of the lithosphere, (3) thermal structure of slabs in the upper mantle, and (4) velocity of slabs in the upper mantle". These are "a priori constraints".

4.9 Line 509: I think I understood that lateral viscosity variations (due to lateral temperature variations) are taken into account. However, the viscosity structure is described as "radial" here. Please confirm that I understood properly and/or not. I also remember from previous work using CitcomS that a thin low-viscosity layer was embedded around the subducting slabs to allow them to remain slab-like features during the subduction process. Is it still the case here?

(Remarks on code availability)
I have scanned/read the code but did not have the time to try to run/execute it and check whether it is usable. It looks very thorough. Much effort has been spent preparing it and, most likely, it will be very useful to the community.

Referee #2

(Remarks to the Author)
A-F:

Dhungana and Flament present a new addition to the debate on the origin of the Great Unconformity, focused on mantle-derived dynamic topography. I can't say I'm convinced that the relatively modest effects of dynamic topography as considered here can entirely explain the extent and magnitude of missing strata across the Great Unconformity, which often extend to times well before that considered in the modelling here. Even so, these processes could certainly represent a meaningful piece of the puzzle, particularly in the context of the termination and preservation of the unconformity. In that context, I would consider the current manuscript a valid addition
to the current debate about the origin of the Great Unconformity. Indeed, I suspect that no single current proposed "cause" (any of my own proposals included) can entirely explain all the available data regarding the GU. While perhaps less satisfying, a combination of causes is certainly not out of the question however, and mantle processes as considered here are a likely enough player.

Assuming the dynamic topography calculations herein are correct (which I will not attempt to assess), I think they could add to our understanding of the pattern of sedimentation through time more generally and how that interacted with the Great Unconformity more specifically. Laurentian subsidence around the start of the Cambrian, as predicted by the model herein, would fit both with the observed geology (i.e., the Sauk sequence) and the characteristic termination of the Great Unconformity thereby.
The lesser (but still present) net subsidence around the start of the Cambrian on most all other continents is also likely broadly consistent with the observed sedimentary record in my view.

If the authors wish to claim to fully explain the entirety of the Great Unconformity,
some much more extensive and quantitative consideration of erosion (potential magnitudes, rates, etc.) would probably be mandatory in my view, with particular note to the fact that uplift (whether by tectonics or dynamic topography) does not itself erode; surface processes erode. However, a claim to a complete standalone explanation for the Great Unconformity is not neither necessary nor necessarily desirable.

G. (references): Yes, appropriate credit given for papers I am familiar with.
H. (clarity and context): No complaints from my perspective.

(Remarks on code availability)

Version 1:

Reviewer comments:

Referee #1

(Remarks to the Author)
Overall, I am satisfied with the revisions that the authors have brought to the manuscript in response to my comments and concerns. Unfortunately, they did not address, in my opinion, the main concern that I expressed, i.e., that the model results depend very strongly on a single plate reconstruction that spans 1 Byr. Recognizing that there is only one such plate reconstruction that can be used to perform their computations while knowing that the reconstruction suffers from large uncertainties, and yet, not providing the reader with an estimate of the uncertainty this brings to the results presented in the paper justifies my concern.

Ideally, and as I suggested in my previous review, the authors should “perturb” the reconstruction and see how the perturbation affects the results. I realize, however, that, in view of the computing cost, this might not be a viable method to demonstrate the sensitivity of the model results to the input to the model.

Alternatively, the authors should come back to the two main plate configurations (or states) that are responsible for the positive then negative DT predicted by the model and lead to the formation and preservation of the Great Unconformity in places where it is observed today. They should consider what are the main features (mostly the location and polarity of the subduction zones, the position of MORs and the divergence rate across them) that drive the mantle flow and discuss whether these features are well constrained in the Meredith et al (2021)’s reconstruction. This could easily be added in a supplementary section where each feature is discussed in terms of its control on the mantle flow and the constraints we have on their existence, location, polarity and rate.

As pointed by the authors, there is a radical change in mantle flow patterns between 640 and 440 Ma. Are the surface features driving this major reorganization well constrained? This transition is also thought to be the expression of a change in mantle dynamics (Cawood and Howkesworth, 2014) at the end of the ‘boring billion’. Do the authors see this transition in their model results, i.e., from reduced plate tectonic activity to subduction-driven mantle flow?

I strongly believe that the results presented in this manuscript are likely to have a much greater impact if these points are addressed and comfort the reader that the findings are strongly rooted in facts and not just models.

As stated above, I am, otherwise, satisfied with the response of the authors to my other comments/suggestions.

Cawood, P. A. & Hawkesworth, C. J. Earth’s middle age. Geology 42, 503–506, 2014

(Remarks on code availability)

Open Access This Peer Review File is licensed under a Creative Commons Attribution 4.0 International License, which permits use, sharing, adaptation, distribution and reproduction in any medium or format, as long as you give appropriate credit to the original author(s) and the source, provide a link to the Creative Commons license, and indicate if changes were made.
In cases where reviewers are anonymous, credit should be given to 'Anonymous Referee' and the source.
The images or other third party material in this Peer Review File are included in the article’s Creative Commons license, unless indicated otherwise in a credit line to the material. If material is not included in the article’s Creative Commons license and your intended use is not permitted by statutory regulation or exceeds the permitted use, you will need to obtain permission directly from the copyright holder.
To view a copy of this license, visit https://creativecommons.org/licenses/by/4.0/

We are thankful to the reviewers for their helpful and constructive comments. Our replies are inserted in blue in this document. Line numbers are provided in blue with respect to the clean version of the revised manuscript, and in purple with respect to the version of the manuscript with tracked changes; these line numbers may be changed as the editorial manager produces pdf files.

Referee #1:

Review of "The Deep Earth Origin of the Great Unconformity" by S. Dhungana and N. Flament

This manuscript reports a very interesting finding linking the Great Unconformity to the large-scale, long-term evolution of the Earth's mantle and the resulting dynamic topography. Using a mantle flow model driven by plate motions and boundaries derived from a deep-time plate reconstruction model, the authors show that only a few areas of the present-day continental surface present the uplift/subsidence history (caused by dynamic topography) conducive to the formation and preservation of the Great Unconformity. Interestingly, these areas coincide with the present locations where this feature has been documented. Furthermore, the authors also show that their model predicts erosion/sedimentation patterns that are (for the most part) consistent with a global atlas of preserved sediment flux.

1. General assessment

The argument developed by the authors is very compelling. It starts with a plate reconstruction model that spans 1 Byr of Earth's history that is used to drive a global mantle flow model, which, in turn, is used to compute dynamic topography. A cluster analysis is then used to show that only a few continental areas have experienced the optimum dynamic topography scenario to produce an unconformity between 920-420 Ma and preserve it afterwards. The authors show that these areas coincide with those where the Great Unconformity is best observed/preserved, i.e., Western Laurentia, Baltica, North China, and Siberia.

I read this manuscript with great enthusiasm and believe it contains material that definitely deserves to be published. The authors took great care in assessing the sensitivity of the results to the model parameters (initial condition, viscosity, and density structure) and used a robust and objective means to compare the predictions to observations (the cluster analysis).

There is no doubt that the material presented in this manuscript is of interest to Nature readership. It is likely to have a very large impact. And I therefore recommend its publication.

Thank you for this positive feedback.

2. Concern

However, I have an important concern about the findings: from what is presented and explained in the manuscript (the manuscript itself, the method section, and the extended data section), it is clear that the patterns of the mantle flow (and thus the patterns and history of dynamic topography) are entirely driven by the assumed plate reconstruction over the last 1 Byr. Unfortunately, the sensitivity of the model results to the assumed plate reconstruction has not been assessed.

We have clarified on (L. 493–497; L. 791–795) that tectonic reconstructions with continuously closing polygons are required in the approach of Bower et al. (2015) to compute velocities. There is currently only one such tectonic reconstruction (Merdith et al., 2021).

In the mantle flow model, surface velocities are imposed, as well as the position of plate boundaries, and the flow induced by subducting slabs. This explains why the results (dynamic topography predictions) are only mildly dependent on the viscosity/density structure and initial conditions. For most, the flow is not dynamical (i.e., driven by internal density variations) but a direct consequence of the imposed boundary conditions. We can see from the model results (Figure 2) and as explained in the text, that positive dynamic topography arises above a BLOB and negative dynamic topography arises in regions surrounded by subduction zones. The BLOB is, in turn, generated as a return flow to the imposed subducting slabs.

We agree with this assessment.

This indicates that the solution of the flow model is strongly dependent on the assumed plate reconstruction, i.e. the location of subduction zones that strongly affects the position of subducting slabs that are the main drivers of the flow and the location of mid-ocean ridges (MORs) that fully controls the thermal structure of the oceanic lithosphere. There exist, however, many alternate plate reconstruction scenarios that could/should be tested. Showing how the results depend on the assumed plate reconstruction would offer a much more convincing argument that the Great Unconformity is indeed produced by dynamic topography. From what is presented here and in the manuscript describing the plate reconstruction model (Merdith et al., 2021), it is very difficult to assess its uncertainty. Merdith et al. (2021) themselves state: "This study is intended as a first step in the direction of a detailed and self-consistent tectonic reconstruction for the last billion years of Earth history". I am, in particular, worried about the uncertainty on the location of MORs and, to a degree, of the subduction zones.

We have added a statement acknowledging the large uncertainty of the tectonic reconstruction (L. 229–230; L. 361–362).

I realize that a large amount of work and computations were needed to arrive at the results presented in this manuscript. But could the authors perform similar computations with different plate reconstructions or with a similar plate reconstruction but "perturbed" within the uncertainty of the geological constraints? If this is not feasible, and in view of the fact that the pattern of dynamic topography can be relatively easily "guessed" from the geometry of the plate boundaries (see my comment above), the authors should be able to assess and present whether alternate plate reconstruction models are likely (or not) to produce similar patterns of dynamic topography. This would be a very worthwhile and instructive exercise that would strengthen the authors' findings (or not).

We now state that testing the sensitivity of the model to the tectonic is a challenge (L. 230–233; L. 362–365).

We have added two new twin cases (M2 and M3) across which the reference frame was varied (no-net-rotation or 'NNR' and paleomagnetic or 'PMAG') to assess the sensitivity of

the results to a perturbation of the reconstruction. In model M3, Laurentia was located above a mantle downwelling at 640 Ma (L. 208–213 and Extended Data Figure 9; L. 328–345). This result illustrates that the solution of the mantle flow model cannot be inferred from any tectonic reconstructions because the velocity field is required to create a mantle frame of reference.

Here is a table mapping the models presented in the original and revised manuscripts:

**Revised manuscript**	**Original manuscript**
M1	M6
M2	–
M3	–
M4	M5
M5	M4
M6	M2
–	M1
–	M3

I believe such a "test" is needed, especially in view of the authors' concluding remark that: "A new opportunity is opening in Earth system modelling to unravel the geodynamic environments of the evolution of complex life by coupling billion-year reconstructions of Earth's evolving deep interior and landscapes to the evolution of global sediment fluxes, sea water chemistry, and diversification of life forms." If indeed, one should consider the reconstructed mantle flow presented here as a basis for further studies, it needs (a) to be better stated that the model is strongly controlled by the assumed plate reconstruction model and (b) to be shown how sensitive the mantle flow model is to that particular plate reconstruction model.

We have broadened the final statement to: 'Future work could investigate the effects of continental-scale dynamic uplift and subsidence on erosion, sedimentation, sea water chemistry, and their consequences for the diversification of life forms.' (L. 290–292; L. 462–464).

3. Suggestion

I note that the discussion (rightly) focuses on how the model explains the conditions for the formation of the Great Unconformity (its extent, its timing, and preservation). It would also be very interesting to understand why such a feature is relatively rare in Earth's history. Is it due to a peculiar distribution of continental masses above the convective mantle? Or is it a consequence of an evolution of convective flow regime at the end of the so-called "boring billion" (period of reduced plate tectonic activity)? Or is it a question of preservation? I believe the authors have all the elements necessary to add a short paragraph in their discussion section that would give the manuscript a greater impact.
I hope the authors will find these comments/suggestions constructive.

We now discuss reasons that could explain why extensive unconformities appear to be rare in a paragraph in which we clarified that dynamic uplift could drive significant glacial erosion (L. 282–285; L. 454–457).

4. Readability and minor points

I found this manuscript relatively well written and fairly pleasant to read. However, a few points need to be clarified or improved as discussed in some of the minor points below. I also note that to fully understand the methodology used, and its implications on the findings, requires a thorough inspection of the method section, the extended data section, and several key references. Although the findings reported here have great potential to be published in a broad journal like Nature, this manuscript could really benefit from a longer format.

4.1: Lines 20-22: This sentence is a repetition of lines 15-18.

We have removed this sentence, which made it possible to mention glacial erosion (L. 18–20; L. 18–20) and possible future work (L. 23–24; L. 23–24) in the abstract.

4.2: Line 34. It is not clear where the number 1200 Myr comes from and what it refers to: is it the maximum extent of the Great Unconformity at a given place on Earth? It seems unlikely that a single process would be responsible for an unconformity that would last this long.

We agree and have removed ‘1,200 Myr’.

4.3 Figure 1: As far as I can tell, this figure is not referred to in the text of the manuscript. It is not clear what it represents as an unconformity is defined as a feature that corresponds to a lack of sedimentation (or removal of sediment). So to what correspond the "oldest sedimentary deposits of the Great Unconformity": are they at the base of the unconformity (or the oldest sediment on top of it) or do they correspond to sediments deposited during the great unconformity (in which case they show the locations of where the unconformity is not)? In any case, I do not think that this is a very good "opening" figure for the article. I would try to have a more direct expression of what is and where is observed the Great Unconformity.

We have added conceptual diagrams to explain what the Great Unconformity is, and kept the original panel that shows where the Great Unconformity can be inferred to exist. We have modified the caption to clarify how the spatial distribution of the Great Unconformity is inferred (L. 37 and L. 58–63; L. 46 and 74–78).

4.4 Line 79: That buoyancy and viscosity variations deeper than 350 km depth only are considered here implies that any source of dynamic topography nearer the surface is neglected, including any "blobbing" or "delamination" of parts of the lithosphere. This should be made clearer here. There are many examples of long-lasting, relatively large features such as the subsidence of the Congo Basin that have been explained with a near-surface source (Downey and Gurnis, 2009, for example).

We have clarified that any source of dynamic topography nearer the surface is neglected and added the suggested reference (L. 83–84; L. 115–116).

4.5 Figure 2: It is unfortunate that the label for Laurentia (LAU) is partly obscured by other labels in several of the panels. Please correct as Laurentia is a key element of this figure.

We have adjusted the position of the label for Laurentia (LAU) to make it clearly visible.

4.6 Figure 3: Panel f is very small. I am not sure how useful it will be once (potentially) reduced to publication size.

We wonder if this could be linked to the resolution of the figures in the version of the manuscript that went out for review. We could adjust the figure if required, and we will provide the figure as a vector graphic.

4.7: Lines 198-201. Interestingly, there is also evidence for strong subsidence and uplift of the Slave Craton during the Phanerozoic (Ault et al., Tectonics, 2013), which contradicts this statement.

We have mentioned that the inferred continental vertical motions are 'averaged in time and space by *k*-means clustering' (L. 269; L. 431) and added a reference to Ault et al. (2013) (L. 272; L. 444).

4.8 Lines 464-466: I do not think that the term "assimilated" is correct here. The model is driven by plate kinematics, plate boundaries, and the assumed positions of subduction zones and MORs. As stated in Bowers et al. (2015): "The methods consist of four constraints determined a priori from a plate history model: (1) plate velocities, (2) thermal structure of the lithosphere, (3) thermal structure of slabs in the upper mantle, and (4) velocity of slabs in the upper mantle". These are "a priori constraints".

We agree and have changed 'assimilated' to 'imposed' (L. 497 and L. 508; L. 795 and L. 842).

4.9 Line 509: I think I understood that lateral viscosity variations (due to lateral temperature variations) are taken into account. However, the viscosity structure is described as "radial" here. Please confirm that I understood properly and/or not.

Viscosity varies laterally with temperature and composition, as well as with depth via pressure dependence and layering. We have deleted the misleading word 'radial'.

I also remember from previous work using CitcomS that a thin low-viscosity layer was embedded around the subducting slabs to allow them to remain slab-like features during the subduction process. Is it still the case here?

A thin low-viscosity layer is not embedded around subducting slabs. We have clarified that the thermal structure of subducting slabs is imposed down to 350 km as described in Bower et al. (2015) (L. 493–497; L. 791–795).

Referee #1 (Remarks on code availability):

I have scanned/read the code but did not have the time to try to run/execute it and check whether

it is usable. It looks very thorough. Much effort has been spent preparing it and, most likely, it will be very useful to the community.

Thank you for this feedback.

****

Referee #2:

A-F:

Dhungana and Flament present a new addition to the debate on the origin of the Great Unconformity, focused on mantle-derived dynamic topography. I can't say I'm convinced that the relatively modest effects of dynamic topography as considered here can entirely explain the extent and magnitude of missing strata across the Great Unconformity, which often extend to times well before that considered in the modelling here. Even so, these processes could certainly represent a meaningful piece of the puzzle, particularly in the context of the termination and preservation of the unconformity. In that context, I would consider the current manuscript a valid addition to the current debate about the origin of the Great Unconformity. Indeed, I suspect that no single current proposed "cause" (any of my own proposals included) can entirely explain all the available data regarding the GU. While perhaps less satisfying, a combination of causes is certainly not out of the question however, and mantle processes as considered here are a likely enough player.

We have clarified that 'sustained positive dynamic topography throughout Cryogenian times (Fig. 3d) would have resulted in significant erosion because glacial erosion rates exceed fluvial erosion rates by an order of magnitude and can operate at lower elevations' (L. 279–280; L. 451–452).

Assuming the dynamic topography calculations herein are correct (which I will not attempt to assess), I think they could add to our understanding of the pattern of sedimentation through time more generally and how that interacted with the Great Unconformity more specifically. Laurentian subsidence around the start of the Cambrian, as predicted by the model herein, would fit both with the observed geology (i.e., the Sauk sequence) and the characteristic termination of the Great Unconformity thereby.
The lesser (but still present) net subsidence around the start of the Cambrian on most all other continents is also likely broadly consistent with the observed sedimentary record in my view.

If the authors wish to claim to fully explain the entirety of the Great Unconformity, some much more extensive and quantitative consideration of erosion (potential magnitudes, rates, etc.) would probably be mandatory in my view, with particular note to the fact that uplift (whether by tectonics or dynamic topography) does not itself erode; surface processes erode. However, a claim to a complete standalone explanation for the Great Unconformity is neither necessary nor necessarily desirable.

We agree and have clarified the important role of glacial erosion in the abstract (L. 18–20; L. 18–20) and discussion (L. 276–282; L. 448–454).

G. (references): Yes, appropriate credit given for papers I am familiar with.
H. (clarity and context): No complaints from my perspective.
****

We are thankful to the referee for their helpful and constructive comments. Our replies are inserted in blue in this document. Line numbers are provided in blue with respect to our clean version of the revised manuscript, and in purple with respect to the version of the manuscript with tracked changes.

Referee #1 (Remarks to the Author):

Overall, I am satisfied with the revisions that the authors have brought to the manuscript in response to my comments and concerns. Unfortunately, they did not address, in my opinion, the main concern that I expressed, i.e., that the model results depend very strongly on a single plate reconstruction that spans 1 Byr. Recognizing that there is only one such plate reconstruction that can be used to perform their computations while knowing that the reconstruction suffers from large uncertainties, and yet, not providing the reader with an estimate of the uncertainty this brings to the results presented in the paper justifies my concern.

Ideally, and as I suggested in my previous review, the authors should "perturb" the reconstruction and see how the perturbation affects the results. I realize, however, that, in view of the computing cost, this might not be a viable method to demonstrate the sensitivity of the model results to the input to the model.

In addition to the computational cost to compute mantle flow models, creating perturbed tectonic reconstructions and associated input files represents a significant amount of work.

Alternatively, the authors should come back to the two main plate configurations (or states) that are responsible for the positive then negative DT predicted by the model and lead to the formation and preservation of the Great Unconformity in places where it is observed today. They should consider what are the main features (mostly the location and polarity of the subduction zones, the position of MORs and the divergence rate across them) that drive the mantle flow and discuss whether these features are well constrained in the Meredith et al (2021)'s reconstruction. This could easily be added in a supplementary section where each feature is discussed in terms of its control on the mantle flow and the constraints we have on their existence, location, polarity and rate.

We now discuss the subduction zones that drive the modelled mantle flow in a supplementary section. The constraints on the existence, location, polarity and convergence rate of subduction zones are listed in the new Supplementary Table 1, and their locations and convergence rates are shown in the new Supplementary Video 2 for the period between 740 Ma and 440 Ma, which encompasses the two main plate configurations shown in Figure 2 (640 Ma and 440 Ma). We mentioned in the main text that the subduction history surrounding Baltica and North China is particularly uncertain during the formation of the Great Unconformity (L. 216–217; L. 231–232).

As pointed by the authors, there is a radical change in mantle flow patterns between 640 and 440 Ma. Are the surface features driving this major reorganization well constrained?

We now mention in the main text that a difference between the two tectonic configurations is the absence of subduction zones south of 50ºS at 640 Ma (L. 94; L. 96), and the presence of subduction zones at high latitude in both hemispheres at 440 Ma (L. 99–100; L. 101–102).

This transition is also thought to be the expression of a change in mantle dynamics (Cawood and Howkesworth, 2014) at the end of the 'boring billion'. Do the authors see this transition in their model results, i.e., from reduced plate tectonic activity to subduction-driven mantle flow?

Cawood and Hawkesworth (2014) argued for a transition at 750 Ma, which is earlier than the reorganisation observed between 640 Ma and 440 Ma. The presented models, in which plate motions and subduction zones are imposed, are not well suited to investigate changes in the style of tectonics and subduction as the solid Earth cooled down.

We have added a reference to Müller et al. (2022) (L. 96–97; L. 98–99) who identified a relevant period of fast plate motion and trench motion between 600 Ma and 320 Ma in the reconstruction of Merdith et al. (2021).

I strongly believe that the results presented in this manuscript are likely to have a much greater impact if these points are addressed and comfort the reader that the findings are strongly rooted in facts and not just models.

As stated above, I am, otherwise, satisfied with the response of the authors to my other comments/suggestions.

Cawood, P. A. & Hawkesworth, C. J. Earth's middle age. Geology 42, 503–506, 2014.
